# Advancing Stem Cell Models of Alpha-Synuclein Gene Regulation in Neurodegenerative Disease

**DOI:** 10.3389/fnins.2018.00199

**Published:** 2018-04-09

**Authors:** Desiree A. Piper, Danuta Sastre, Birgitt Schüle

**Affiliations:** Parkinson's Institute and Clinical Center, Sunnyvale, CA, United States

**Keywords:** alpha-synuclein, *SNCA*, transcriptional regulation, induced pluripotent stem cells, Parkinson's disease, Dementia with Lewy bodies

## Abstract

Alpha-synuclein (*non A4 component of amyloid precursor, SNCA, NM_000345.3*) plays a central role in the pathogenesis of Parkinson's disease (PD) and related Lewy body disorders such as Parkinson's disease dementia, Lewy body dementia, and multiple system atrophy. Since its discovery as a disease-causing gene in 1997, alpha-synuclein has been a central point of scientific interest both at the protein and gene level. Mutations, including copy number variants, missense mutations, short structural variants, and single nucleotide polymorphisms, can be causative for PD and affect conformational changes of the protein, can contribute to changes in expression of alpha-synuclein and its isoforms, and can influence regulation of temporal as well as spatial levels of alpha-synuclein in different tissues and cell types. A lot of progress has been made to understand both the physiological transcriptional and epigenetic regulation of the alpha-synuclein gene and whether changes in transcriptional regulation could lead to disease and neurodegeneration in PD and related alpha-synucleinopathies. Although the histopathological changes in these neurodegenerative disorders are similar, the temporal and spatial presentation and progression distinguishes them which could be in part due to changes or disruption of transcriptional regulation of alpha-synuclein. In this review, we describe different genetic alterations that contribute to PD and neurodegenerative conditions and review aspects of transcriptional regulation of the alpha-synuclein gene in the context of the development of PD. New technologies, advanced gene engineering and stem cell modeling, are on the horizon to shed further light on a better understanding of gene regulatory processes and exploit them for therapeutic developments.

## Introduction

Alpha-synuclein (*non A4 component of amyloid precursor, SNCA, chr4q21-22, NM_000345.3, MIM #163890*) was the first gene in which a causative mutation for Parkinson's disease (PD) was discovered in 1997 (Polymeropoulos et al., 1997; Nussbaum, 2017). The detection of alpha-synuclein protein as a constituent of Lewy bodies further strengthened the role of alpha-synuclein in PD and dementia with Lewy bodies (DLB) as a central player in the pathogenesis of neurodegeneration (Spillantini et al., 1997; Goedert et al., 2017). It is now well-established that rare point mutations and large genomic multiplications of the *SNCA* gene can cause autosomal-dominant parkinsonism which can present itself as a wide range of clinical and histopathological features including typical PD (Langston et al., 2015), Parkinson's disease dementia (PDD), DLB (Beyer et al., 2009), multiple system atrophy (MSA) (Jellinger and Lantos, 2010), or even fronto-temporal dementia (FTD) (Polymeropoulos et al., 1997; Krüger et al., 1998; Singleton et al., 2003; Chartier-Harlin et al., 2004; Farrer et al., 2004; Ibáñez et al., 2004; Zarranz et al., 2004; Nishioka et al., 2006; Fuchs et al., 2007a; Deng and Yuan, 2014).

Genetic variants in the non-coding region of the *SNCA* gene, including single nucleotide polymorphisms (SNPs) were discovered by association and genome wide association studies (GWAS) (Nalls et al., 2014; Campelo and Silva, 2017) and small structural variants increase the risk for developing PD (Chiba-Falek, 2017), however the underlying mechanisms are still under investigation as will be reviewed herein.

The mere overexpression of wildtype alpha-synuclein in patients with *SNCA* multiplications is sufficient to cause parkinsonism and point mutations in the *SNCA* gene seem to increase alpha-synuclein inclusion formation or its interaction with acidic phospholipids suggesting that alpha-synuclein, when mutated, causes neurodegeneration and parkinsonism via a toxic gain-of-function mechanism (Rajagopalan and Andersen, 2001; Collier et al., 2016).

Interest in alpha-synuclein as a therapeutic target for PD has been growing not only due to the genetic link, but also because of the pathology of alpha-synuclein spreading through the nervous system and the cell-to-cell propagation of aggregated alpha-synuclein. These observations have led to consensus that lowering the alpha-synuclein content and/or eliminating toxic alpha-synuclein species in cells could be the key to slowing, reversing, or even preventing the disease and such alpha-synuclein lowering therapeutic strategies are currently being developed (Bergström et al., 2016; Brundin et al., 2017).

While advancements of genetic technologies have greatly increased our understanding of the genetics of the *SNCA* gene, alpha-synuclein aggregation, interaction, and post-translational modification has been intensively studied over the last two decades, there is still a gap in current knowledge about transcriptional and epigenetic regulation of *SNCA* gene and how it contributes to PD and related alpha-synucleinopathies. This has been in part due to the paucity of suitable methods, but also because of lack of models and complexity of the modeling of temporal and cell-specific transcriptional regulation of gene expression.

In addition, recent advances in our understanding of stem cell biology, nuclear reprogramming, and genome engineering offer new avenues for disease modeling of PD given the challenges of animal models for progressive neurodegenerative aspects of the disease. Nuclear reprogramming has been successful in modeling virtually any adult somatic tissue type using different delivery protocols of integrating (Takahashi et al., 2007) or non-integrating viruses (Yang et al., 2008), episomal vectors (Su et al., 2014), excisable vectors (Woltjen et al., 2009), mRNA (Mandal and Rossi, 2013), protein (Seo et al., 2017), or chemical compounds (Hou et al., 2013; Kimura et al., 2015; Silva et al., 2015). Regarding PD, patient-specific induced pluripotent stem cells (iPSCs) lay the foundation for differentiation into the tissue-type of interest, i.e., mid-brain dopaminergic neurons. As a result, there is a great excitement and an intensive effort within the PD community to derive iPSCs from patients with genetic and sporadic forms of PD to model disease and use these cells for exploring disease mechanisms and for drug discovery (Schüle et al., 2009; Imaizumi and Okano, 2014). Specifically, Mendelian forms of PD have been of particular interest including SNCA, which is the focus of this review, leucine-rich repeat kinase 2 (LRRK2), glucosidase, beta, acid (GBA), PTEN-induced putative kinase 1 (PINK1), or PARKIN (reviewed in Hartfield et al., 2012; Torrent et al., 2015). Furthermore, genome engineering allows for generation of isogenic cell lines that only differ by the introduced change in the genome which allows for the correction of mutations or introduction of genetic variants to understand their functional consequences (Hockemeyer and Jaenisch, 2016). Mutant forms of these nucleases can be utilized for complex transcriptional modulation, such as Clustered Regularly Interspaced Short Palindromic Repeats interference (CRISPRi; Du and Qi, 2016; Mandegar et al., 2016).

A working hypothesis for the importance of studying the physiological regulation of the *SNCA* gene is that a subtle to moderate overexpression of alpha-synuclein due to one or more genetic risk factors (or in combination with environmental triggers) over many decades can either predispose or even cause the neurodegenerative changes similar to *SNCA* gene multiplications. Neurons subjected to higher, non-physiological levels of alpha-synuclein might be more likely to be damaged by misfolding and aggregation of this protein, eventually leading to neuronal cell death.

This review focuses on genetic variants in the *SNCA* gene that predispose to PD and on how transcriptional regulation directed by the non-coding and epigenomic elements within the *SNCA* gene can lead to PD and neurodegeneration. Furthermore, we highlight emerging new technologies and stem cell models for studying gene regulation, e.g., CRISPRi screens will allow for rapid advancements in our understanding of transcriptional regulation of the non-coding genome and epigenome.

## Multiplications of the *SNCA* gene lead to rapid progressive parkinsonism

### SNCA genomic triplications

**Figure 1:**
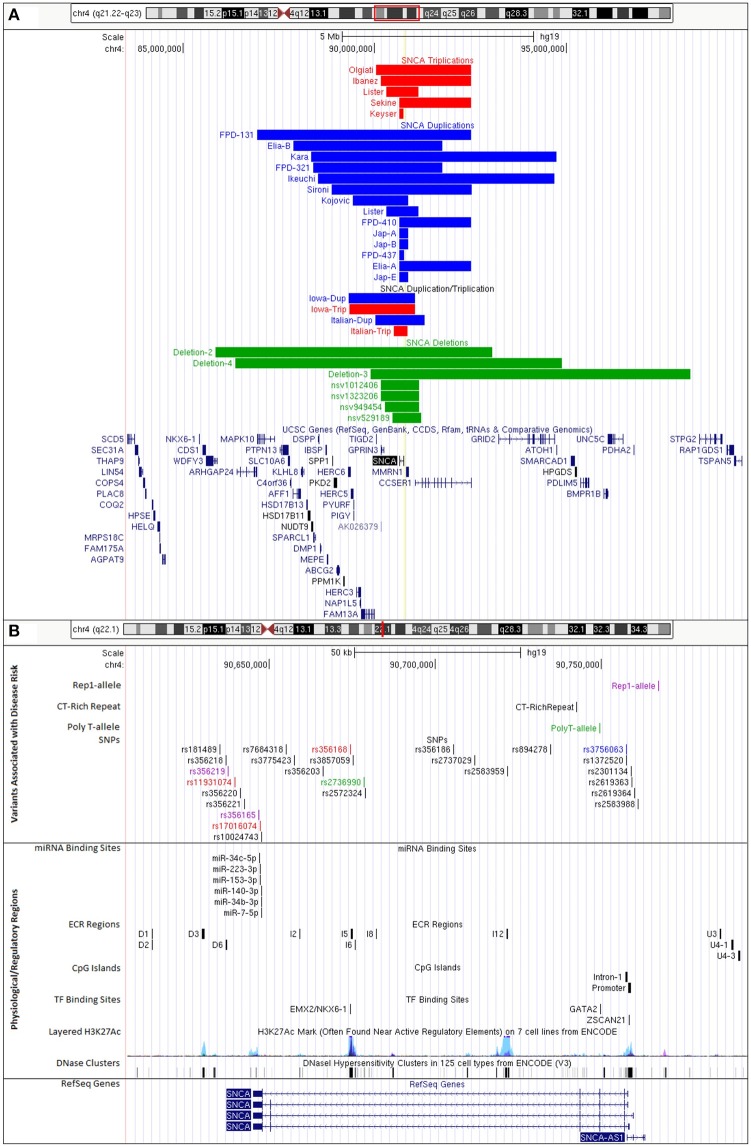
UCSC Genome Browser custom tracks for PD-risk associated variants and regulatory regions impacting SNCA expression. **(A)** CNVs of SNCA locus on chromosome 4q21.23-q22.3 (GRCh37/hg19, chr4:84,239,011-98,739,011). Colors indicate gene copy numbers. Red: *SNCA* CNV triplications; blue: *SNCA* CNV duplications; combination: *SNCA* triplication (red) and duplication (blue); green: *SNCA* CNV deletions. IDs given to tracks were either based on family identifiers from literature or are first author's last name of publication where case/family has been reported. **(B)** Disease variants and regulatory regions of the SNCA genomic locus on chromosome 4q22.1 (GRCh37/hg19, chr4:90,608,984-90,793,984) (details in Supplementary Tables 2–4). Colors indicate functional changes related to variants. Red: total mRNA expression; blue: affects *SNCA* splice-isoform; green: *SNCA* methylation; pink: multiple associated functions. Additional tracks include microRNA and transcription factor binding sites, CpG islands, and integrated regulation from ENCODE (Layered H3K27Ac and DNase Clusters). (Supplementary Table 5 lists all positions to build custom tracks in UCSC Genome Browser).

Copy number variants (CNVs) of the *SNCA* genomic locus on chromosome 4q21 were first discovered in 2003 in a family with parkinsonism now termed the Iowa kindred (Singleton et al., 2003). The mutation is a genomic triplication of the *SNCA* gene and adjacent genes resulting in a size of ~1.7 Mb. Patients with an *SNCA* triplication carry four functional copies of the *SNCA* gene, three copies from the mutant allele and one copy from the wildtype allele, resulting in a 2-fold overexpression of alpha-synuclein mRNA and protein. We recently performed a high-resolution comparative genomic hybridization array and determined that this *SNCA* triplication was derived in two steps with an underlying region of a slightly larger duplication (Zafar et al., 2018; Figure 1A, Supplementary Table 5).

### SNCA genomic duplications

In addition, there are also families with *SNCA* genomic duplications (total of three *SNCA* gene copies) of chromosome 4q21 (Konno et al., 2015). The length of the genomic regions varies (Ross et al., 2008; Kara et al., 2014; Figure 1A), there are additional cases presenting a combination of duplications and triplications (Ferese et al., 2015). We mapped size and location of published cases with *SNCA* triplication and duplications (Figure 1A) and also identified through database searches CNV deletions of the *SNCA* locus. Interestingly, these CNV deletion cases were from large cohorts of children with intellectual disability/developmental delay, multiple congenital anomalies, and/or autism (Miller et al., 2010; Vulto-van Silfhout et al., 2013; Coe et al., 2014; Duyzend et al., 2016; Figure 1A). A common primary mechanism underlying the generation of such microdeletions/duplications is non-allelic homologous recombination (NAHR) that give rise to a number of disorders with such reciprocal rearrangements of specific chromosomal regions (Hastings et al., 2009; Watson et al., 2014). The SNCA/4q21 locus has been primarily recognized with various forms of parkinsonism as a duplication/triplication, but detailed clinical presentations of SNCA/4q21 microdeletions are pending.

Clinically, the *SNCA* duplications generally present with typical PD, whereas *SNCA* triplications have an earlier onset of motor symptoms (34.5 ± 7.9 years for triplication vs. 47.2 ± 10.6 years for duplication) and in addition present more frequently with cognitive decline at an earlier age (39.6 ± 5.5 years for triplication vs. 56.5 ± 9.6 years for duplication) (Book et al., 2017). The increasing severity of the clinical and histopathological phenotype with the number of *SNCA* gene copies clearly points to gene dosage effect for the *SNCA* gene.

### SNCA genomic triplication stem cells models replicate known disease mechanisms

The *SNCA* genomic triplication was also the first mutation for which patient pluripotent stem cells were derived and characterized (Byers et al., 2011; Devine et al., 2011; Table 1 Supplementary Table 1). Undifferentiated patient-derived iPSCs carrying the *SNCA* triplication show a 2-fold increase in SNCA mRNA and protein expression according to the genomic copy number. Although alpha-synuclein is more highly expressed in differentiated neurons, the ratio of 2-fold increased expression of alpha-synuclein compared to sibling controls is maintained. The *SNCA* triplication neurons are more prone to oxidative stress, and show changes on neuronal maturation which is illustrated by a lower expression of tyrosine hydroxylase, the rate limiting enzyme for dopamine synthesis (Oliveira et al., 2015). Also, patient-derived neuroprecursors while showing normal cellular and mitochondrial morphology, exhibit substantial changes in growth, viability, cellular energy metabolism, and stress resistance when challenged by starvation or toxicant challenge. However, these phenotypic changes were reversible upon *SNCA* knockdown (Flierl et al., 2014). Another study has investigated the cell-to-cell transfer for alpha-synuclein and describes that the secretion of alpha-synuclein in the medium is increased in the *SNCA* triplication neurons and furthermore, secreted alpha-synuclein is taken up by co-cultured mouse neuroblastoma cells (Reyes et al., 2015).

**Table 1:** Human iPSC models with mutations and genetic variants in *SNCA* gene elicit specific molecular and cellular phenotypes.

**References**	**iPSC Source and clonal lines**	**Mutation type**	**Phenotype in human iPSC model**
***SNCA*** **CNV**			
Byers et al., 2011	Skin fibroblasts: 1 patient (*SNCA* triplication from Iowa kindred, 42 yrs male), 1 control (46 yrs female, mutation-negative sibling)	CNV	*SNCA*-tri and control lines had similar pluripotency marker expression and neuronal differentiation patterns. Alpha-synuclein levels are higher in *SNCA*-tri iPSCs and neurons compared to controls. There is a 1.5- to 4-fold increased expression of oxidative stress and protein aggregation-related genes in *SNCA*-tri cultures.
Devine et al., 2011	Skin fibroblasts: 1 patient (*SNCA* triplication from Iowa kindred, 55 yrs, female), 1 control (first degree relative)	CNV	SNCA-tri and control fibroblasts do not have detectible protein levels of alpha-synuclein. Alpha-synuclein protein detected in all iPSC-derived neurons. Elevated *SNCA* expression found in SNCA-tri iPSCs which increased with differentiation. SNCA-tri -derived neurons showed 2-fold increase of *SNCA* mRNA compared to controls. For SNCA paralogous genes, *SNCG* expression was significantly lower in SNCA-tri neurons but *SNCB* was unchanged.
Flierl et al., 2014	iPSCs 1 patient (*SNCA* triplication, 42 yrs male), 2 healthy controls (46 yrs female sibling, 61 yrs old male Byers et al., 2011)	CNV	SNCA-tri NPCs had normal cellular and mitochondrial morphology but altered growth, viability, cellular energy metabolism, and stress resistance. Knockdown of alpha-synuclein by shRNA reversed phenotypic alterations.
Oliveira et al., 2015	iPSC-derived neural progenitors (NPCs): 1 patient (*SNCA* triplication, 42 yrs male), 2 controls (unaffected sister, 46 yrs; unrelated healthy control, 62 yrs, male Byers et al., 2011; Flierl et al., 2014)	CNV	SNCA-tri overexpresses alpha-synuclein and expression increases during *in vitro* neuronal differentiation. *SNCA*-tri neurons fail to develop complex networks and showed reduced neurite outgrowth. TH+ cell number was lower in *SNCA*-tri than control. Over-expression of alpha-synuclein impairs neuronal maturation. *SNCA*-tri neurons presented lower neuronal activity. Genes associated with neuronal differentiation and signal transduction were down regulated in *SNCA*-tri.
Reyes et al., 2015	Skin fibroblasts: 1 patient (*SNCA* triplication), 1 control (mutation negative family member Devine et al., 2011)	CNV	Differentiated neurons from *SNCA*-tri patient secrete higher levels alpha-synuclein compared to control neurons. Five-day co-cultures SNCA-tri neurons and N2a cells with restricted cell-to-cell contact showed alpha-synuclein puncta around and within the N2a cells.
Heman-Ackah et al., 2017	iPSCs: patient with *SNCA* triplication (ND34391G iPSCs, NINDS/Coriell Institute), 1 control (NCRM-5, NIH CRM), 13 CRISPR-edited isogenic clones	CNV	Alpha-synuclein mRNA and protein levels were reduced in CRISPR-edited isogenic iPSC clones (two functional SNCA gene copies). SNCA-tri has little effect on neuronal differentiation based on RNA-Seq. Ninety-fold overexpression of *SNCA* mRNA in SNCA-tri neurons were restored in isogenic controls. The three branches of UPR were upregulated in AST neurons. SNCA-tri showed ER stress phenotype, induction of IRE1a/XBP1 axis [unfolded protein response (UPR)] and UPR activation.
Mittal et al., 2017	iPSC-derived NPCs: 1 patient (*SNCA* triplication, 42 yrs male), 1 healthy control (46 yrs female sibling Flierl et al., 2014)	CNV	Beta-adrenoreceptor agonist clenbuterol reduces alpha-synuclein expression by 20% in SNCA-tri NPCs. Clenbuterol reduces mitochondria-associated superoxide in SNCA-tri and positively affects viability when exposed to rotenone. Alpha-synuclein downregulation by beta-adrenoreceptor agonists was shown to be mediated by a decrease in H3K27 acetylation in promoter and intron 4 enhancers of the *SNCA* gene.
***SNCA*** **POINT MUTATIONS**			
Chung et al., 2013	iPSCs (*SNCA*, p.A53T (female, AAO 49 yrs; Golbe et al., 1996) and *SNCA* triplication Byers et al. (2011), 1 male control (BG01)	CNV and Point mutation	In yeast, nitrosative stress is caused by alpha-synuclein and contributes to toxicity. There is also increased nitric oxide in A53T cortical neurons compared to corrected neurons. *SNCA* p.A53T alpha-synuclein leads to ERAD dysfunction. NAB2, an N-arylbenzimidazole, activates Rsp5/Nedd4 pathway and reduced nitric oxide levels in *SNCA* p.A53T neurons. NAB2 improves forward protein trafficking through ER in SNCA-tri neurons.
Soldner et al., 2011	Skin fibroblasts 1 patient (*SNCA* p.A53T mutation, Golbe et al., 1996 and Supplementary Table 1 Chung et al., 2013,) BG01 and WIBR3 hESCs	Point mutation	Several pairs of ZFN-isogenic hiPSC/hESCs were generated and characterized for neuronal differentiation: hESC—hESC^SNCAA53T/wt^, hESC—hESC^SNCAE46K/wt^, hiPSC SNCA p.53T—hiPSC corrected. *SNCA* p.A53T was inserted into *SNCA* gene via ZFN without drug selection. Increased efficiency of introducing a second mutation (*SNCA* p.E46K) via single-stranded oligodeoxynucleotides into hESCs. *SNCA* wild-type sequence containing donor vector and ZFNs genetically corrected *SNCA* p.A53T mutation in patient-derived hiPSCs.
Ryan et al., 2013	2 isogenic pairs: iPSCs (Soldner et al., 2011 *SNCA* p.A53T and paired mutation ZFN-corrected clone); hESC (BG01) line and paired ZFN-induced SNCA p.A53T mutation	Point mutation	iPSC-derived dopaminergic neurons from *SNCA* p.A53T carrier show alpha-synuclein aggregation resembling Lewy body-like pathology. *SNCA* p.A53T mutant neurons display variations in mitochondrial machinery and an increase in mitochondrial toxin susceptibility. ROS/RNS abundance leads to changes of MEF2C in *SNCA* p.A53T neurons.
***SNCA*** **RISK VARIANTS AND GENE REGULATION**			
Soldner et al., 2016	hiPSC line derived from fibroblast AG20446 (male, PD, 57 yrs) and 2 hESCs from Whitehead Institute Center for Human Stem Cell Research and NIH (WIBR3, BG01)	SNV and SSV	Generation of CRISPR-modified isogenic hESC allelic panels for *SNCA* gene risk variants rs356168 and NACP-Rep-1. CRISPR insertion of G-allele at rs356168 results in increased expression of *SNCA*. Sequence-specific binding of TFs EMX2 and NKX6-1 represses intron 4 enhancer activity, modulating *SNCA* expression. Allelic series of NACP-Rep1 (genotypes 257/261, 259/261 261/261 263/261) did not show expression differences for alpha-synuclein.
Heman-Ackah et al., 2016	Skin fibroblasts: a patient with *SNCA* triplication (ND34391G, iPSCs from NINDS/Coriell Institute), 1 control (NCRM-5, RUDCR Infinite Biologics)	*SNCA* gene regulation	Binding affinity between different sgRNAs and relative position to the TSS are critical for CRISPRi. dCas9 can be used for gene expression manipulations and gene contributions of neurodegenerative disease. CRISPR/dCas9-KRAB and TSS2-1 sgRNA expression reduced endogenous alpha-synuclein mRNA levels in SNCA-tri iPSC-derived neurons by 40%.
Tagliafierro et al., 2017	IPSCs from healthy patient (GM23280, Coriell Repository), iPSCs from *SNCA*-tri patient (ND34391, NINDS Repository)	miRNA expression	Differentiation into two different neuronal cell types, midbrain dopaminergic and cholinergic neurons, were developed. MiR-7-5p, miR-153-3p, and miR223-3p had higher levels in dopaminergic neurons while miR-140-3p was only slightly increased in cholinergic neurons. SNCA-tri miR-7-5p levels in neurons were 10-fold decreased compared to control neurons, other miRNAs showed similar trends as in control neurons.

iPSC-derived neurons from the *SNCA* triplication were also utilized for drug screening and repurposing. Using an unbiased screen targeting endogenous alpha-synuclein gene expression, b2-adrenoreceptor (b2AR) agonists were discovered as a regulator of the alpha-synuclein gene (*SNCA*). b2AR ligands were shown to downregulate *SNCA* transcription through inhibition of histone 3 lysine 27 acetylation by about 20% (Mittal et al., 2017). These findings were confirmed in neuroprecursors derived from the induced pluripotent stem cells of a patient carrying the *SNCA* triplication which demonstrated that b2AR agonist clenbuterol could normalize *SNCA* expression and reduce alpha-synuclein protein, and ameliorate mitochondrial function (Mittal et al., 2017).

Three additional studies should be mentioned that utilized the *SNCA* triplication line from Devine et al. (2011). The first study developed a CRISPR inhibition system to downregulate alpha-synuclein. The authors found one specific guide RNA that downregulated alpha-synuclein by 50% at the mRNA and protein level in healthy controls and neurons from the *SNCA* triplication carrier (Heman-Ackah et al., 2016).

Generating isogenic iPSCs that only differ by the introduced mutation is believed to greatly reduce biological noise. Two studies show independently the CRISPR/Cas9 targeting of exonic regions of the *SNCA* gene (exon 2 or exon 4) and create panels of isogenic lines with various functional copies of the *SNCA* gene (Heman-Ackah et al., 2017; Zafar et al., 2017). Interestingly, comparative expression analysis of neuronal cultures from the parental *SNCA* triplication and a 2-copy knockdown presented with an ER stress phenotype, marked by induction of unfolded protein response (UPR) and leading to terminal UPR activation (Heman-Ackah et al., 2017).

These patient-iPSC studies in *SNCA* genomic triplications emphasize that this *in vitro* cellular model reflects many aspects of neurodegeneration as described in human tissues and other models (Table 1, Supplementary Table 1).

## Missense mutations in the coding region of the *SNCA* gene

Polymeropoulos et al., identified the first causative mutation for PD, a missense mutation in exon 3 of the SNCA gene (p.A53T, NM_000345.3:c.157G>A; Polymeropoulos et al., 1997) which ignited the era of genetics in PD. However, the frequency of mutations in the *SNCA* gene remains low, at about 0.5% in familial and sporadic cases (Deng and Yuan, 2014). There are only a few point mutations that are considered clearly causative or pathogenic, p.A30P (NM_000345.3:c.88G>C; Krüger et al., 1998, 2001; Seidel et al., 2010), p.E46K (NM_000345.3:c.136G>A; Zarranz et al., 2004), p.H50Q (NM_000345.3:c.150T>G; Appel-Cresswell et al., 2013), p.G51D (NM_000345.3:c.152G>A; Kiely et al., 2013, 2015; Lesage et al., 2013), p.A53E (NM_000345.3: c.158C>A; Pasanen et al., 2014; Martikainen et al., 2015), and p.A53V (NM_000345.3: c.158C>T; Yoshino et al., 2017).

Interestingly, while most of the *SNCA* missense mutations present with typical Lewy body Parkinson's disease (reviewed in Langston et al., 2015), some of these point mutations show additional Parkinson-plus clinical features. In a recent case report, two unrelated patients with *SNCA* p.A53T mutation presented with early-onset frontal-dysexecutive dysfunction with apathy and resembling frontotemporal dementia (FTD) followed by motor symptoms of PD. An MRI showed marked frontal, parietal and temporal, neocortical atrophy, and atrophy in the mesial temporal lobe. No autopsy data are presented (Bougea et al., 2017). The *SNCA* p.A53E was reported in a patient with atypical PD starting at the age of 36 which came to autopsy at the age of 60 with fulminant alpha-synuclein pathology characterized as MSA and PD (Kiely et al., 2013; Pasanen et al., 2014). Patients with *SNCA* p.G51D mutations show clinical signs of early-onset rapidly progressive levodopa responsive parkinsonism, but can also present with pyramidal signs and severe cognitive impairment and hallucinations (Lesage et al., 2013; Tokutake et al., 2014; Kiely et al., 2015). In a case with *SNCA* p.A51D mutation, unusual neuropathological findings were described, including fine, diffuse cytoplasmic inclusions containing phospho-alpha-synuclein at position 129 in superficial layers of the cerebral cortex and entorhinal cortex together with severe neuronal loss in the substantia nigra and locus ceruleus (Lesage et al., 2013).

*In vitro* experiments of mutant alpha-synuclein shows that several of the SNCA point mutations increase the propensity of alpha-synuclein to form fibrils, including p.A53T, p.E46K, and p.H50Q (Conway et al., 1998; Narhi et al., 1999; Greenbaum et al., 2005; Appel-Cresswell et al., 2013; Ghosh et al., 2013; Proukakis et al., 2013). On the other hand, the pathogenic point mutations p.A30P, p.G51D, and p.A53E seem to attenuate the fibrillation rate of alpha-synuclein (Li et al., 2001; Fares et al., 2014; Ghosh et al., 2014; Pasanen et al., 2014), which suggests that fibrillation rate is not directly involved in PD pathogenesis and other kinetics such as oligomerization should be tested and pursued (Ghosh et al., 2017).

### SNCA p.A53T point mutation and genetically engineered isogenic controls in human stem cells models

Using zinc-finger technology, two point mutations, p.A53T, NM_000345.3:c.157G>A and p.E46K (NM_000345.3:c.136G>A) were genetically engineered into human pluripotent stem cells and the p.A53T mutation was genetically corrected (Soldner et al., 2011). Derived clonal lines were karyotypically normal and maintained neuronal differentiation potential (Soldner et al., 2011). Phenotypic differences were presented in a follow-up publication for the p.A53T point mutation which show increased thioflavin T staining which detects amyloid aggregates increased alpha-synuclein phospho 129 immunofluorescence which represents a pathological form of alpha-synuclein found abundantly in Lewy bodies (Ryan et al., 2013). Also, mitochondrial function was impaired as shown by an increase in ROS species (Ryan et al., 2013).

In iPSC-derived cortical neurons from a patient with the *SNCA* p.A53T mutation and the *SNCA* triplication nitrosative stress, accumulation of ER-associated degradation (ERAD) substrates and ER stress was observed as an early pathogenic phenotype (Chung et al., 2013). Based on the discovery in a yeast screen, the authors identified a small molecule which activated E3 ubiquitin ligase neural precursor cell expressed developmentally down-regulated protein 4 (Nedd4) and rescued the molecular phenotype in human iPSC-derived cortical neurons (Chung et al., 2013; Table 1, Supplementary Table 1).

## Repeat variants in the *SNCA* gene predispose to PD

### Rep-1 allele

Probably the most studied polymorphism in the 5′ region of the *SNCA* gene is the non-A-beta component of Alzheimer disease amyloid, precursor (NACP)-Rep1 polymorphism of the *SNCA* promoter. The Rep1 dinucleotide repeat is located 9.8 kb upstream of the transcriptional start site of the *SNCA* gene and exhibits five alleles of different sizes. Some studies showed an association of the Rep1 allele with PD (Krüger et al., 1999; Farrer et al., 2001; Tan et al., 2003; Pals et al., 2004; Mellick et al., 2005; Hadjigeorgiou et al., 2006), but other studies could not reproduce this association (Parsian et al., 1998; Khan et al., 2001; Spadafora et al., 2003). There could be multiple reasons for non-replication such as differences in populations, sample size, methodological differences for standardization of sampling and measurements. Only a large collaborative effort for 2,692 cases and 2,652 controls showed the association of the long Rep-1 allele with PD (odds ratio, 1.43) (Maraganore et al., 2006; Figure 1B).

The Rep-1 repeat acts as a modulator of *SNCA* transcription and shows a 4-fold increase in promoter activity (Touchman et al., 2001). The longest repeat (263bp allele, risk allele for PD) resulted in a 2.5-fold increase in luciferase activity over the shortest repeat. The 261bp allele (major allele) showed only a 1.5-fold increase over the shortest repeat, whereas the 259 bp allele increased expression by 3-fold (Chiba-Falek et al., 2003). Furthermore, in human brain tissue from 228 PD cases and 144 normal cases, the protective 259 bp (homozygous) allele presented with a 40–50% *SNCA* mRNA reduction in the temporal cortex and substantia nigra (Linnertz et al., 2009).

### Modeling of a genetically engineered rep1 allelic series in human pluripotent stem cells

Recent work in human embryonic stem cell (ESC)/iPSC-derived neuronal models in which the four haplotypes (alleles 257/261, 259/261, 261/261, and 263/261) were genetically engineered by CRISPR, the increase in *SNCA* mRNA expression was not confirmed in 25-day neuronal cultures from two different human embryonic stem cell lines (WIBR3 and BGO1; Soldner et al., 2016). However, when comparing these data with the data from human brain, only human brains that carried the homozygous protective allele (259/259) showed a significant downregulation of SNCA mRNA expression (Linnertz et al., 2009). Alternatively, the lack of differentiation expression of the Rep-1 alleles in the model could be due to the neuronal subtypes that were neuronally differentiated. The iPSC-neuronal cultures primarily consist of glutamatergic neurons and *SNCA* gene might not be regulated through the Rep-1 allele like the effect seen in human brain regions from the frontal cortex where no change in *SNCA* mRNA expression was detected in any of the Rep-1 haplotypes (Linnertz et al., 2009). In addition, it could also have a technical explanation with respect to primer design and quantification of the various *SNCA* isoforms and transcripts (Chiba-Falek, 2017; Table 1, Supplementary Table 1).

### *SNCA* CT-rich haplotype

Recently, an intronic polymorphic CT-rich region was identified in the *SNCA* gene through a genome-wide screen for short structural variants (SSV) (Saul et al., 2016; Chiba-Falek, 2017). The SSV evaluation system focuses on GWAS regions and prioritizes SSVs in close proximity to the GWAS signal based on repeat context, conservation tracks, epigenetic histone marks, transcription factor binding sites, and miRNA binding sites (Saul et al., 2016). The intron 4 *SNCA* CT-rich SSV has four different haplotypes and the risk haplotype is associated with Lewy body pathology in Alzheimer disease. In addition, the *SNCA* CT-rich risk haplotype leads to a higher mRNA expression of *SNCA* in human brain. This suggests that the *SNCA* CT-rich haplotype acts as an enhancer region in the *SNCA* gene (Lutz et al., 2015; Figure 1B). An iPSC model for haplotypes of this variant is underway (Chiba-Falek, 2017).

### Intron 2 poly-T

An intronic poly-T polymorphism upstream of exon 3 (rs149886412) comprises three alleles (5T, 7T, and 12T). The most common genotypes are 7T/7T (47.0%) and 7T/12T (34.9%), whereas less frequent alleles are 5T/7T and 12T/12T (8.7% each), and 5T/12T is very rare (0.7%) (Beyer et al., 2007). Interestingly, the length of the poly-T polymorphism is associated with expression of the *SNCA* splice isoform *SNCA* 126. *SNCA*126 levels are lower in blood from carriers with the 5T allele and increased when 12T allele is present (Beyer et al., 2007). Comparing different age groups for the presence of the poly-T allele, the shorter allele 5T/7T was more frequent at a younger age, whereas the 12T allele was more frequent with increasing age in control subjects (Beyer et al., 2007). The length of the poly-T allele could affect the distance to splicing enhancers or silencer, thus modulating alternative splicing for *SNCA* exon 3 (Figure 1B).

This polymorphism is another candidate for generating an isogenic panel of pluripotent stem cells similar to Soldner et al. for the Rep-1 allele (Soldner et al., 2016).

## GWAS hits in the *SNCA* gene increase PD risk

More than 50 studies have been conducted investigating whether certain SNPs within the *SNCA* gene are associated with parkinsonism either as case-control studies or within GWAS (Mueller et al., 2005; Mizuta et al., 2006; Ross et al., 2007; Winkler et al., 2007; Nalls et al., 2014; Campelo and Silva, 2017). Many SNPs have been identified and confirmed throughout the *SNCA* gene encompassing the 5′ region/promoter, intron 4, and 3′ region/3′UTR (Figure 1B). Exploring the effects and functional relevance of these SNPs is becoming a new focus of research and we are beginning to unravel the underlying phenomena and mechanisms that lead to increased disease risk for PD and related alpha-synucleinopathies. These functional effects of SNPs can be categorized as (i) changes in levels of total alpha-synuclein mRNA/protein expression, (ii) changes in *SNCA* isoform expression, (iii) changes in DNA methylation of *SNCA* CpG oligodeoxynucleotide regions, and (iv) changes in transcription factor binding/enhancer/repressor sites or miRNA binding sites. Out of 25 PD-associated SNPs, seven have been reported to influence alpha-synuclein levels, however, some findings are controversial (Supplemental Table 2). Six SNPs within intron 4 and 3′ region were studied for total alpha-synuclein mRNA levels in post-mortem brain samples, blood, or *in vitro* with variable findings for alpha-synuclein expression (Fuchs et al., 2007a, 2008; Westerlund et al., 2008; Sotiriou et al., 2009; Mata et al., 2010; Hu et al., 2012; Rhinn et al., 2012; Cardo et al., 2014; Glenn et al., 2017).

One study evaluated whether disease-associated SNPs affect *SNCA* splicing. There are four *SNCA* isoforms that can arise due to alternative splicing of exons 3 and 5: alpha-synuclein 98 (missing exon 3 and 5), alpha-synuclein 112 (missing exon 5), and alpha-synuclein 126 (missing exon 3) in addition to the full length alpha-synuclein 140 (Beyer and Ariza, 2013). Interestingly, alpha-synuclein 126 has been shown to be reduced in DLB whereas isoform 112 is overrepresented in DLB (Beyer et al., 2006). Three SNPs (rs2736990, rs356165, rs356219) facilitate expression of the alpha-synuclein 112 isoform in frontal cortex. The location of the SNPs were predicted to lie within splice enhancer domains which could explain the effect on 112 isoform over total alpha-synuclein 140 expression (McCarthy et al., 2011).

A SNP in intron 1 (rs3756063, CpG19) which is located within the differentially methylated region of the *SNCA* gene, has been consistently described to mitigate hypomethylation in PD cases in blood and brain (Pihlstrøm et al., 2015; Schmitt et al., 2015; Wei et al., 2016).

Conceptually, the alteration of a transcription factor binding site by a SNP is an attractive hypothesis to study. One publication describes a 3′ region SNP (rs356219, A-allele, protective) to preferentially bind the transcription factor yin yang 1 (YY-1). While alpha-synuclein expression was unchanged, the antisense non-coding RNA RP11-115D19.1 was stimulated by YY1 overexpression, whereas knockdown of RP11-115D19.1 increased alpha-synuclein expression.

In addition to gene engineering the Rep1 allele in human iPSCs, Soldner et al. also identified a SNP in intron 4 which is located within an enhancer region (Soldner et al., 2016). By CRISPR/Cas9 mediated genome editing, an allelic series of this SNP was generated in human pluripotent stem cells and the G-allele showed increase in alpha-synuclein expression in neuroprecursor cells and differentiated neurons. Furthermore, in electrophoretic mobility shift assays (EMSA) SNP-dependent binding of empty spiracles homeobox 2 (EMX2) and homeobox protein NKX6-1 with preference for the protective lower expressing A-allele at rs356168 was observed. This is the first study, that used iPSC modeling to interrogate functional effects of PD associated SNPs. The same SNP was subsequently tested for changes in *SNCA* expression in frontal and temporal cortex of a cohort of 134 healthy individuals. That study concluded the opposite effect for the G-allele with an ~20% decrease in alpha-synuclein mRNA in individuals homozygous for the G-allele (Glenn et al., 2017).

While it is exciting to witness the advancements toward a functional understanding of disease-associated non-coding SNPs, more work is needed to successfully address the complexity of transcriptional regulation in a disease context.

While it is necessary to directly study target tissue, in this case with a human brain, in which transcriptional changes are expected, there are at this point limitations to this approach which may influence the results, such as sample size per genotype, confounding factors which add biological noise, post-mortem interval, mixture of neuronal and glial cell populations, and finally the fact that in post-mortem tissue from patients with neurodegenerative disease, there is usually a moderate to severe neuronal loss of the cell type of interest.

Human stem cell models might be able to mitigate some of these challenges. Gene engineering of human pluripotent stem cells allow for specific generation of genotypes and allelic series of genotypes which should reduce experimental variability. Differentiation protocols allow for high yield of specific cell types which can be further enriched by cell sorting. Lastly, these models can be further manipulated by toxins to address environmental triggers and gene-environment interactions.

## Methylation in the intron 1 is related to PD pathogenesis

DNA methylation at CpG sites is considered an epigenetic regulation mechanism to fine-tune transcriptional regulation of genes (Li and Zhang, 2014). In general, DNA methylation is considered to repress gene expression. New findings also include hydroxymethylation which seems to be specific for gene regulation in the central nervous system (Wen and Tang, 2014).

The *SNCA* gene contains a large CpG island in the promoter region comprising 70 CpGs, which does not seem to be differentially methylated in PD. However, there is another smaller CpG region in intron 1 of the *SNCA* gene that has been identified to be methylated in cancer cell lines (Matsumoto et al., 2010).

Studies of DNA methylation in PD can be divided into global DNA hydroxy-/methylation (Desplats et al., 2011; Masliah et al., 2013; Stöger et al., 2017), specific methylation of *SNCA* intron 1 in human brain regions (Matsumoto et al., 2010; de Boni et al., 2011, 2015; Desplats et al., 2011; Guhathakurta et al., 2017) and peripheral tissues (Song et al., 2014; Tan et al., 2014; Eryilmaz et al., 2017; Funahashi et al., 2017; Supplemental Table 3).

Overall, the *SNCA* intron 1 region is hypomethylated with 0.5–3% methylation in brain (de Boni et al., 2011; Guhathakurta et al., 2017) and 6–10% methylation in peripheral blood cells (Song et al., 2014; Tan et al., 2014) and comparison between cohorts of PD and controls show controversial results, possibly due to experimental techniques, small sample size, or mixture of tissue types in samples. None of the studies showed an effect of *SNCA* expression due to hypomethylation of the region.

The studies for global methylation are similarly controversial in their findings with one study found hypomethylation in a PD sample set, whereas another study did not report a difference in global methylation (Desplats et al., 2011; Masliah et al., 2013). Nevertheless, hydroxymethylation was reported to be lower in the cerebellum in PD samples vs. controls (Stöger et al., 2017).

Human pluripotent stem cell models would present a unique model to interrogate DNA methylation changes introduced by environmental toxins or stressors and cell types of interest can be selected or enriched by cell sorting. On the other hand, CRISPR/Cas9 techniques can selectively methylate CpG sites via deactivated Cas9 nuclease and catalytic domain of the DNA methyltransferase DNMT3A to experimentally study effects of DNA methylation on gene expression (Vojta et al., 2016).

## *SNCA* gene regulation by transcription factors

Transcription factors are a group of proteins that bind to specific DNA sequences or motifs and are critical for the regulation of gene expression in a temporal and spatial fashion throughout life (Mitchell and Tjian, 1989). The transcriptional regulation of the *SNCA* gene by trans-acting transcription factors is not entirely understood. One of the first reports of transacting factors regulating the *SNCA* gene was poly-(ADP-ribose) transferase/polymerase-1 (PARP-1), a DNA-binding protein and transcriptional regulator, which was shown to bind to the Rep-1 repeat by EMSA, CHIP, and mass spectrometry. When PARP-1 was inhibited, it increased *SNCA* mRNA levels in SH-SY5Y cells (Chiba-Falek et al., 2005). In luciferase assays, PARP-1 binding to the NACP-Rep1 element reduced the transcriptional activity of the REP1/*SNCA* promoter construct (Chiba-Falek et al., 2005).

In *in vitro* studies in SH-SY5Y cells, overexpression of transcription factor CCAAT/enhancer-binding protein β (C/EBPβ) also increases alpha-synuclein expression and eight C/EBPβ interaction motifs CCAAT were predicted within the *SNCA* promoter region (Gomez-Santos et al., 2005).

Next, it was shown that the hematopoietic transcription factor GATA-binding factor 1 (GATA-1) activates *SNCA* transcription in GIE-ER-GATA-1 cells and GATA-1 occupies a specific region in the *SNCA* promoter (Scherzer et al., 2008). The neuronal counterpart GATA-2 is highly expressed in dopaminergic neurons and knockdown of GATA-2 in SH-SY5Y cells resulted in a decrease of alpha-synuclein expression (Scherzer et al., 2008).

Transcription factor zinc finger and SCAN domain containing 21 (ZSCAN21) which is expressed in different brain areas, regulates alpha-synuclein expression (Dermentzaki et al., 2016). Depending on the cell type and maturation level, ZSCAN21 can have antagonistic effects on transcription of alpha-synuclein. Silencing of ZSCAN21 leads to an increase in alpha-synuclein expression in mature cortical cultures of rats. On the contrary, when ZSCAN 21 is knocked down in neurosphere stem cell cultures, alpha-synuclein expression is reduced, but no alterations are observed in postnatal and adult hippocampus. When ZSCAN21 is overexpressed in cortical neurons only *SNCA* mRNA is increased, but not alpha-synuclein protein expression which suggests that ZSCAN21 is not a master regulator and additional regulatory mechanisms are involved (Dermentzaki et al., 2016).

Brenner et al. (2015) was able to detect GATA2 and C/EBPβ in frontal cortex, cingulate gyrus and medulla oblongata and ZSCAN, although expressed at low levels, was detected by IP assays. *In silico* analysis revealed 17 putative binding sites for these transcriptions factors. By ChIP analysis, only two predicted transcription factor binding sites could be confirmed for ZSCAN21 in intron 1 GATA-2 for intron 2 of the *SNCA* gene (Figure 1B, Supplemental Table 4). No direct interaction for C/EBPβ was determined (Brenner et al., 2015).

Recently, p53 was shown to have a binding site CATG in murine *SNCA* promoter at positions −970 to −967, and a feedback loop between alpha-synuclein and p53 has been postulated where upon depletion of p53 alpha-synuclein is downregulated (Duplan et al., 2016; Alves da Costa et al., 2017). Follow-up studies in human models should confirm these very interesting findings that could link neurodegeneration and cancer (Alves da Costa et al., 2017).

Human pluripotent stem cell models are ideal to interrogate these questions due to the human genetic background, ability to derive specific cell types and ease of *in vitro* studies with relevant phenotypes.

## Non-coding RNAs in the *SNCA* region regulating *SNCA* expression

MicroRNAs (miRs, miRNAs) are a class of single-stranded non-coding RNAs of 18–22 nucleotides and play a key post-transcriptional role in gene expression. miRNAs function by base-pairing with complementary sequences of the target mRNA molecule most commonly within the 3′ untranslated (UTR) region. This binding activates the RNA-induced silencing complex (RISC), leading to downregulation of the gene by cleavage of target mRNAs by RISC, translational repression, or mRNA decapping and decay (Karnati et al., 2015).

There is now growing evidence that alterations of miRNAs play a role in the pathogenesis of PD (Qiu et al., 2015). The first publication that linked miRNA regulation to PD found that miR-133b is specifically expressed in post-mitotic midbrain dopaminergic neurons and regulates maturation and function via negative feedback loop by paired-like homeodomain transcription factor Pitx3. In human midbrain samples expression of miR-133b is significantly reduced in PD cases (Kim et al., 2007). To date, additional miRNAs have been implicated PD including some that have shown to directly bind to and negatively regulate alpha-synuclein expression (Recasens et al., 2016). Several studies confirmed that both miR-7 and miR-153 are highly expressed in the brain and have been shown to bind to the 3′ untranslated region of *SNCA* and can downregulate *SNCA* expression levels. Interestingly, these two miRNAs have an additive effect on *SNCA* downregulation (Junn et al., 2009; Doxakis, 2010; Figure 1B). In addition, miR-34b and 34c as well as miR-140 and miR-223 have been shown *in vitro* assays or have been predicted to bind to the SNCA 3′UTR (Lim and Song, 2014; Kabaria et al., 2015; Wang et al., 2015; Tagliafierro et al., 2017).

### Differential miRNA expression in iPSC-derived midbrain and cortical neurons

The first study of miRNA assessment in human iPSC models was published by Tagliafierro et al. (2017). Differentiation into two different neuronal cell types, midbrain dopaminergic neurons and cholinergic neurons, were developed to understand regulation of miRNAs in neurons that exhibit histopathological features of PD and DLB. MiR-7-5p, miR-153-3p, and miR223-3p showed higher levels in dopaminergic neurons while miR-140-3p was slightly increased in cholinergic neurons (Tagliafierro et al., 2017) from *in vitro* cultures of a healthy control. When comparing miRNA levels in neuronal cultures from a *SNCA* genomic triplication, a 10-fold decrease in expression levels of miR-7-5p was shown compared to neurons from a healthy iPSC control, while other miRNAs showed similar trends as in control neurons (Tagliafierro et al., 2017). It will be interesting to understand why miR-7-5p levels are downregulated and how this might contribute to neurodegeneration of dopaminergic neurons (Table 1, Supplementary Table 1).

## Discovery of novel regulatory elements by combining *in silico* comparison with *in vitro* validation

Non-coding conserved genomic regions (ncECRs) within a certain gene, or even up to several hundred kilobases away, can serve as enhancers, silencers, or modifiers to ensure the accurate temporal and spatial expression of a gene by recruiting transcription factors that bind to them.

Comparative genomics is based on the prioritization of a genomic region by searching for highly conserved non-coding sequences between several species to identify potential functional regulatory elements. The rationale behind this approach is that functional or regulatory sequence elements are under a selection pressure and do not diverge as rapidly as “neutral” sequences. By comparative analyses between different species (e.g., human-mouse) or multiple comparisons including fish, non-coding conserved regions can be recognized, which could harbor gene regulatory function (Ahituv et al., 2004; Bird et al., 2006). Examples that this comparative genomics approach is useful for showing the identification of functional variants in non-coding regulatory regions in otherwise mutation-negative families (Marlin et al., 1999; Lettice et al., 2003; Sabherwal et al., 2007), understanding of regulatory elements within disease genes such as Ret proto-oncogene (RET) and Methyl-CpG binding protein 2 (MECP2) (Grice et al., 2005; Liu and Francke, 2006), or identification of a functional SNP responsible for Warfarin toxicity (Rieder et al., 2005). In a pair-wise comparison of the *SNCA* genomic region, 32 evolutionary conserved DNA sequences with high homology to mouse were identified and 11 conserved sequence elements showed an increase or reduction of luciferin activity which indicates cis-regulatory function on gene expression which need to be further evaluated to be linked to alpha-synuclein expression (Sterling et al., 2014).

## New high-throughput technologies to dissect transcriptional regulation

Even though many mechanisms of transcriptional regulation of the *SNCA* gene at the physiological level and in context of disease have been studied, many areas still remain controversial and need further evaluation and confirmation. Human pluripotent stem cell models could become a new benchmark for success as they can be more tightly controlled via new gene engineering technologies allowing the creation of allelic series of human stem cell lines with defined genotypes. In addition, novel high-throughput technologies could pave the way to identify regulatory elements at a much larger scale.

Recent advancements in technology such as CRISPR gene manipulation allows us now to directly interrogate genomic regions in the context of endogenous genes and in the target tissue which makes the results easier to interpret. Two groups identified non-coding regulatory elements by high-throughput CRISPR screens (Fulco et al., 2016; Sanjana et al., 2016). These pooled CRISPR screens utilize CRISPR interference which can alter chromatin state through a Krüppel associated box (KRAB) effector domain fused to catalytically dead Cas9 (dCas9), but do not introduce permanent mutations. This allows for the characterization of the regulatory effects of up to one megabase of genomic sequence at a given locus (Sanjana, 2016; Wright and Sanjana, 2016) and several thousand guide RNAs can be simultaneously tested. Fulco et al. assessed 1 Mb sequence around myelocytomatosis oncogene cellular homolog (MYC) and GATA-binding factor 1 (GATA1) and identified 9 enhancers (Fulco et al., 2016), whereas Sanjana et al. targeted ~700 kb of sequence around the genes neurofibromatosis type 1 (NF1), neurofibromatosis type 2 (NF2), and cullin 3 (CUL3) and found non-coding regions that modulate drug resistance (Sanjana et al., 2016). This CRISPR interference screen might be a tool to identify and confirm regulatory elements in the *SNCA* genomic locus.

To better understand the functional effects of non-coding SNPs, a new pipeline was developed which includes fine-mapping, epigenomic profiling, and epigenome editing and then interrogation for causal function by using genome editing to create isogenic pluripotent stem cell lines (Spisák et al., 2015). A very similar approach was successfully implemented by Soldner et al. (2016) who assessed histone acetylation, generated panel of isogenic lines with series of genotypes, and then tested the expression of the *SNCA* gene (Soldner et al., 2016).

## Combining human pluripotent stem cell models with post-mortem tissues to decipher the role of SNCA transcriptional regulation in neurodegenerative disease

To further advance the field of studying functional effects of disease-associated risk variants, it is critical to contrast and compare different models and tissues. This review focuses on the use of human tissues (primarily brain and blood samples) and how human ESC/iPSC models can complement such case-control series.

Studies of human brain are very valuable, however, there are several challenges when assessing human brain, such as biological differences and confounding factors like age, gender, environmental exposure/lifestyle, disease duration, concomitant disease. Next, circumstances of death, post-mortem interval, preservation methods, and dissections protocols of the tissue vary and influence quality of samples. Since transcriptional regulation is cell type-specific, therefore the assessment of a mixed neuronal population can mask changes in rarer cell types, contrariwise the isolation of cellular subtypes in frozen brain using e.g., laser-capture technique or cell sorting is technically demanding. Lastly, the cell type of interest in a brain area might have been already degenerated during the disease process, thus the target cell type of interest is no longer present.

Human iPSC models on the other hand can address some of the challenges of studying human brain. Human iPSCs have a human genetic background and are derived from patients with or without known disease-causing mutations. These iPSC cultures can be genetically engineered to create isogenic cell lines or allelic series that only differ by the introduced genetic variant thus greatly reducing biological noise. What should be considered when studying *SNCA* transcriptional regulation in human iPSC-differentiated cultures is the temporal manipulation of *SNCA* gene expression or introduction of mutations *in vitro* e.g., by CRISPR. As described for the SNCA CNVs, a CNV deletion resulting in presumably 50% reduction in gene expression can potentially contribute clinically to developmental delay or autism. Depending on the introduction of *SNCA* transcriptional modulation, there might be effects in differentiation efficiency, phenotypic stability and outcomes. Furthermore, these iPSCs can be differentiated into many cell and tissue types of the human body. *In vitro* differentiation protocols are constantly being improved to faithfully mimic the target cell types of interest not only for expression of cell markers, but also for physiological function, e.g., dopaminergic neurons or organoids for PD (Jo et al., 2016; Kirkeby et al., 2017), cholinergic neurons for DLB/Alzheimer-related DLB (Duan et al., 2014), enteric nervous system (Workman et al., 2017), or oligodendrocytes for MSA (Djelloul et al., 2015). Side-by-side comparison of human primary tissues with evolving human iPSC models can inform and complement the current experimental approaches for transcriptional regulation.

## Conclusion

Transcriptional regulation is complex and requires both cis-acting genomic elements as well as trans-acting transcription factors in a temporal and tissue-specific manner. Alterations introduced by mutations or epigenetic changes could disturb this tightly regulated system and lead to diseases such as PD, LBD/Alzheimer-related Lewy body disease and other alpha-synucleinopathies. These diseases share alpha-synuclein protein conformational changes and aggregation as a common denominator; however, the distribution, progression, and severity differ. Understanding the temporal and cell-type specific regulation of alpha-synuclein might provide insights in the development and distribution of alpha-synucleinopathies as the disruption of transcriptional regulation could be the underlying culprit ultimately driving shared pathological processes in these diseases.

## Author contributions

BS, DP, and DS: Contributed to the conception and design of the study; DP: Prepared UCSC custom tracks and reviewed iPSC publications; BS: Wrote the first draft of the manuscript; DP and DS: Wrote sections of the manuscript. All authors contributed to manuscript revision, read, and approved the submitted version.

### Conflict of interest statement

The authors declare that the research was conducted in the absence of any commercial or financial relationships that could be construed as a potential conflict of interest.
