# Supplementary material for: Advancing Stem Cell Models of Alpha-Synuclein Gene Regulation in Neurodegenerative Disease
Source: Front Neurosci. 2018 Apr 9;12:199. doi: 10.3389/fnins.2018.00199 (PMC5900030; doi:10.3389/fnins.2018.00199)
Supplement: Supplementary file 1 [file DataSheet1.pdf]

Supplementary Material

Advancing stem cell models of alpha-synuclein gene regulation in neurodegenerative disease

Desiree A. Piper, Danuta Sastre, Birgitt Schüle*

Parkinson's Institute and Clinical Center, Sunnyvale, CA, U.S.A.

*** Correspondence:** Birgitt Schüle, bschuele@thepi.org

Supplementary Tables:

Supplemental Table 1: Expanded iPSC table for *SNCA* iPSC models

Supplemental Table 2: Functional SSVs/SNPs in the *SNCA* gene

Supplemental Table 3: *SNCA* gene methylation in PD and related disorders

Supplemental Table 4: Transcription factor binding in *SNCA* gene

Supplemental Table 5: Positions/coordinates for UCSC genome browser custom tracks

Supplemental Table 1: Expanded iPSC table for *SNCA* iPSC models

***SNCA* CNV**	
**(Byers et al., 2011)**	
**iPSC Source**	Skin fibroblasts: 1 patient (*SNCA* triplication from Iowa kindred, 42 yrs male), 1 control (46 yrs female, mutation-negative sibling)
**iPSC Reprogramming/Number of clones**	Lentivirus OSKM, 3 patient clones (Trpl8, 17, 43), 3 control clones (Ctrl 1, 2, 3), and H9
**Gene Editing**	N/A
**iPSC Maintenance/Passaging**	On iMEF cells or Matrigel in hESC media /mechanical passaging.
**Neural Induction**	Co-culture with stromal cells, for 16d in 15% KOSR/KO-DMEM. After 16d, media changed to N2 for 2d.
**Neuronal Differentiation/Time**	Neural rosettes dissection, transfer on PO/L for 7d in N2 (+Shh/C24II, +FGF8, +BDNF, +AA). D 35 mechanical passage on PO/L in N2 (+BDNF, +GDNF, +TGF-β, cAMP, AA). Day 50: Final differentiation in N2 (+BDNF, GDNF TGF-β3, cAMP, AA). Total time 50d.
**Phenotype**	*SNCA*-tri and control lines had similar pluripotency marker expression and neuronal differentiation patterns. Alpha-synuclein levels are higher in *SNCA*-tri iPSCs and neurons compared to controls. There is a 1.5- to 4-fold increased expression of oxidative stress and protein aggregation-related genes in *SNCA*-tri cultures.
**(Devine et al., 2011)**	
**iPSC Source**	Skin fibroblasts: 1 patient (*SNCA* triplication from Iowa kindred, 55yrs, female), 1 control (first degree relative)
**iPSC Reprogramming/Number of clones**	Retrovirus OSKM, 8 patient clones, 6 control clones
**Gene Editing**	N/A
**iPSC Maintenance/Passaging**	On iMEF cells in hESC media (KO-DMEM, 20% KSR, NEAA, β-ME, 10ng/ml FGF2) / Mechanical pass.
**Neural Induction**	Monolayer dissociated with Accutase, selection in MEF-CM (+Y27632, +FGF2) on gelatin for 3d, transfer to feeder-free in hESC (KSR +Noggin, + SB4315423, +Dorsomorphin), after 1d +Shh/C24II, +Wnt for 5d.

**Neuronal Differentiation/Time**	Switch to N2B27 for 7d. N2B27 +Shh, +BDNF, +AA, +FGF8). After 3d, mechanical passaging onto PLO! In N2B27 (+AA, +BDNF, +cAMP) for 7d. Total time 23-31d.
**Phenotype**	SNCA-tri and control fibroblasts do not have detectible protein levels of alpha-synuclein. Alpha-synuclein protein detected in all iPSC-derived neurons. Elevated *SNCA* expression found in SNCA-tri iPSCs which increased with differentiation.<br>SNCA-tri -derived neurons showed 2-fold increase of *SNCA* mRNA compared to controls. For SNCA paralogous genes, *SNCG* expression was significantly lower in SNCA-tri neurons but *SNCB* was unchanged.
**(Flierl et al., 2014)**	
**iPSC Source**	iPSCs 1 patient (*SNCA* triplication, 42 yrs male), 2 healthy controls (46 yrs female sibling, 61 yr old male) (Byers et al., 2011)
**iPSC Reprogramming/Number of clones**	Retrovirus OSKM[1], 2 clones for *SNCA* trip, 1 clone each for controls
**Gene Editing**	N/A
**iPSC Maintenance/Passaging**	On iMEF cells + Geltrex in (DMEM/F12, 20% KSO, NEAA, β-ME, FGF2)/Mechanical and enzymatic, Coll/ Disp. Accutase passaging.
**Neural Induction**	EB suspension culture for 4d in EB medium (hESC, -FGF2 +/- Dorsomorphine, SB431542). 2-3d in NIM (DMEM/F12, NEAA, N2, FGF2) feeder free. Manual rosette isolation and feeder free culture with enzymatic passaging in NPC media (Neurobasal, B27, NEAA, FGF2),PSA-NCAM sorting[4] . Total time 7d.
**Neuronal Differentiation/Time**	N/A
**Phenotype**	SNCA-tri NPCs had normal cellular and mitochondrial morphology but altered growth, viability, cellular energy metabolism, and stress resistance. Knockdown of alpha-synuclein by shRNA reversed phenotypic alterations.
**(Oliveira et al., 2015)**	
**iPSC Source**	iPSC-derived neural progenitors (NPCs): 1 patient (*SNCA* triplication, 42 yrs male), 2 controls (unaffected sister, 46 yrs; unrelated healthy control, 62 yrs, male) (Byers et al., 2011; Flierl et al., 2014)
**iPSC Reprogramming/Number of clones**	Retroviral OSKM (1 clone, 2 controls), Lentiviral single vector with OSKM (1 clone)

**Gene Editing**	N/A
**iPSC Maintenance/Passaging**	On iMEF cells in hESC media (DMEM/F12, 20% KSR, NEAA, P/S, β-ME, L-Glu, FGF2) / Mechanical Passage.
**Neural Induction**	iPSC colonies harvested with collagenase IV, transferred to bacterial petri dish, EBs formed in suspension with agitation on rocker in EB media (hESC media without FGF2 w/wo Dor, SB431532) x4d, EBS cultured with agitation in NIM (DMEM/F12, NEAA, L-FLU, N2) for 2-3d, plated on Geltrex. Rosettes in 2-5d in adherent culture. NiPSCs plated (Geltrex) in NiPSCs media (MACS Neuro Medium, MACS NeuroBrew-21, L-Glu, NEAA, P/S, ESGRO (LIF), bFGF) up to 25 passages (accutase at confluency into new dish).
**Neuronal Differentiation/Time**	Incubate cells in DA1 medium (MACS Neuro Medium, MACS NeuroBrew-21, L-Glu, P/S, rh-FGF-8a, smoothened agonist SAG) for 10d. Reseed and incubate for 20d in DA2 medium (MACS Neuro Medium, MACS NeuroBrew-21, L-Glu, NEAA, P/S, rh-GDNF, rh-BNDF, dibutyryl-cAMP. Total time ~42d.
**Phenotype**	SNCA-tri overexpresses alpha-synuclein and expression increases during *in vitro* neuronal differentiation. *SNCA*-tri neurons fail to develop complex networks and showed reduced neurite outgrowth. TH+ cell number was lower in *SNCA*-tri than control. Over-expression of alpha-synuclein impairs neuronal maturation. *SNCA*-tri neurons presented lower neuronal activity. Genes associated with neuronal differentiation and signal transduction were down regulated in *SNCA*-tri.
**(Reyes et al., 2015)**	
**iPSC Source**	Skin fibroblasts: 1 patient (*SNCA* triplication), 1 control (mutation negative family member) (Devine et al., 2011)
**iPSC Reprogramming/Number of clones**	Retrovirus pMXs-OSKM
**Gene Editing**	N/A
**iPSC Maintenance/Passaging**	On SNL feeders in hESC media (KO-DMEM, KSR, L-Glu, NEAA, 2-Merc, P/S, FGF2, Valproate) w/o valproate. Once established, switch to hESC medium with less FGF2.

**Neural Induction**	Timed exposer to LDN193189/SB431542 (day 0-5), Shh/FGF8 (day 1-7), LDN193189 alone (day 5-11), CHIR99021 (day 3-13). Cells grown on matrigel in KSR (DMEM, KSR, L-Glu, β-Me) for 11d. KSR media switched to N2 media on day 5. Day 11 media switched to Neurobasal/B27/L-Glu medium (supp. CHIR [until d13], BDNG, ascorbic acid, GDNF, TGFβ3, dibutyryl cAMP, DAPT) for 9d.
**Neuronal Differentiation/Time**	Cells lifted via accutase and replated on polyornithine/laminin/fibronectin on day 20 in differentiation medium (NB/B27+BDNF, ascorbic acid, GDNF, dbcAMP, TGFβ3, DAPT) until maturation.<br>Total time 20d.
**Phenotype**	Differentiated neurons from *SNCA*-tri patient secrete higher levels alpha-synuclein compared to control neurons. 5-day co-cultures SNCA-tri neurons and N2a cells with restricted cell-to-cell contact showed alpha-synuclein puncta<br>around and within the N2a cells.
**(Heman-Ackah et al., 2017)**	
**iPSC Source**	iPSCs: patient with *SNCA* triplication (ND34391G iPSCs, NINDS/Coriell Institute), 1 control (NCRM-5, NIH CRM), 13 CRISPR-edited isogenic clones
**iPSC Reprogramming/Number of clones**	N/A
**Gene Editing**	Correction of *SNCA* triplication in *SNCA* exon 4 via double-nicking CRISPRs
**iPSC Maintenance/Passaging**	On iMEF cells for 4-day expansion, individual clones manually transferred to Matrigel coated 96-well plates and later expanded into 6 well plates.
**Neural Induction**	iPSCs cultured in Neural Induction Media (SB431542 and Dorsomorphin) for 30d.
**Neuronal Differentiation/Time**	Cell suspension seeded on poly-L-ornithine, day 1 media replaced with Neuronal Differentiation Medium (Neurobasal, B27, GlutaMax, BDNF, GDNF, dbcAMP), media replaced every other day x10-14 d.<br>Total time ~45d.

**Phenotype**	Alpha-synuclein mRNA and protein levels were reduced in CRISPR-edited isogenic iPSC clones (2 functional SNCA gene copies). SNCA-tri has little effect on neuronal differentiation based on RNA-Seq. 90-fold overexpression of *SNCA* mRNA in SNCA-tri neurons were restored in isogenic controls. The three branches of UPR were upregulated in AST neurons. SNCA-tri showed ER stress phenotype, induction of IRE1a/XBP1 axis (unfolded protein response (UPR)) and UPR activation.
**(Mittal et al., 2017)**	
**iPSC Source**	iPSC-derived NPCs: 1 patient (*SNCA* triplication, 42 yrs male), 1 healthy control (46 yrs female sibling) (Flierl et al., 2014)
**iPSC Reprogramming/Number of clones**	N/A
**Gene Editing**	N/A
**iPSC Maintenance/Passaging**	On iMEF cells + Geltrex in (DMEM/F12, 20% KSO, NEAA, β-ME, FGF2)/Mechanical and enzymatic, Coll/ Disp. Accutase passaging.
**Neural Induction**	EB suspension culture for 4d in EB medium (hESC, -FGF2 +/- Dorsomorphine, SB431542). 2-3d in NIM (DMEM/F12, NEAA, N2, FGF2) feeder free. Manual rosette isolation and feeder free culture with enzymatic passaging in NPC media (Neurobasal, B27, NEAA, FGF2),PSA-NCAM sorting[4] . Total time 7d.
**Neuronal Differentiation/Time**	N/A
**Phenotype**	Beta-adrenoreceptor agonist clenbuterol reduces alpha-synuclein expression by 20% in SNCA-tri NPCs. Clenbuterol reduces mitochondria-associated superoxide in SNCA-tri and positively affects viability when exposed to rotenone. Alpha-synuclein downregulation by beta-adrenoreceptor agonists was shown to be mediated by a decrease in H3K27 acetylation in promoter and intron 4 enhancers of the *SNCA* gene.
***SNCA* Point Mutations**	
**(Chung et al., 2013)**	
**iPSC Source**	iPSCs (*SNCA*, p.A53T (female, AAO 49yrs, (Golbe et al., 1996; Soldner et al., 2011)) and *SNCA* triplication (Byers et al., 2011), 1 male control (BG01)
**iPSC Reprogramming/Number of clones**	Lentivirus KOS (inducible, excisable)/ 2 subclones /genotype

**Gene Editing**	Correction of mutation in patient A53T iPSC by ZFN
**iPSC Maintenance/Passaging**	On iMEF cells in hESC medium (DMEM/F12, 15%FBS, 5% KSO, NEAA, β-ME, FGF2) mechanical or enzymatic.
**Neural Induction**	Feeder-free, enzymatic pass (Accutase) in mTESR1. Dissoc. iPSC resuspended and aggregated in DMEM/F12 (1% N2, 1% B27, Noggin, SB431542, Y-27632). D4: EB transfer to feeder-free (Matrigel) FGF2, Dkk1. Day 10 rosettes dissected, enzymatic, replate on PO/L in NPC medium (Neurobasal, FGF2, Y27632) /2 clones/genotype.
**Neuronal Differentiation/Time**	NPCs dissociated enzymatically (Disp.) and replated feeder-free (Matrigel) in Neurobasal Medium (BDNF, GDNF, cAMP). D7-9 cells were replated on PO/L in Neurobasal medium for <5d. Total time 19-20d.
**Phenotype**	In yeast, nitrosative stress is caused by alpha-synuclein and contributes to toxicity. There is also increased nitric oxide in A53T cortical neurons compared to corrected neurons. *SNCA* p.A53T alpha-synuclein leads to ERAD dysfunction. NAB2, an N-arylbenzimidazole, activates Rsp5/Nedd4 pathway and reduced nitric oxide levels in *SNCA* p.A53T neurons. NAB2 improves forward protein trafficking through ER in SNCA-tri neurons.
**(Soldner et al., 2011)**	
**iPSC Source**	Skin fibroblasts 1 patient (*SNCA* p.A53T mutation, (Golbe et al., 1996) and Table S1 (Chung et al., 2013), BG01 and WIBR3 hESCs
**iPSC Reprogramming/Number of clones**	Lentivirus OSKM, (Dox-inducible., Cre-excisable.). 1 clone /genotype
**Gene Editing**	*SNCA* p.A53T and p.E46K gene editing by ZFN, 1 p.A53T "corrected" clone (WIBR-iPS-*SNCA*), 1 p.E46K induced clone (BGO1)
**iPSC Maintenance/Passaging**	On iMEF cells in hESC medium (DMEM/F12, 15% FBS, 5% KSR, NEAA, β-ME, FGF2) / mechanical + enzymatic (Coll/Disp) passaging.
**Neural Induction**	Matrigel cultured iPSCs in MEF-conditioned medium (+FGF2) Coll dissociated. Forced-aggregate formation of EBs, feeder-free suspension culture in A: [1%N2, 4% B27/DMEM:F12] with Y-27632 and Noggin or Dorsomorphin for 1-2d. Transfer to feeder-free culture in A (-Y-27632, +Dkk1, +FGF2). /Multiple clones used for targeting, analysis of 1-4 clones/genotype.

**Neuronal Differentiation/Time**	Neural Rosette formation for 8d in suspension culture with EB medium. At d 8 plated on fibronectin dishes in ITS[10]. After 8d, enzymatic pass onto PO/L in N2 medium plus AA, FGF2, FGF8, Shh for 8d. Terminal differentiation in N2 (- FGF2, FGF8, Shh) for 8d. Mechanical pass. on stromal feeder cells for 14d, followed by 7d in N2 (all 21d with noggin), Neural Rosettes at d21 pass. on PO/L for 16d in N2 medium with Shh, FGF8, BDNF, AA. Final differentiation in N2 plus BDNF, TGF-β3, GDNF, cAMP, AA for 5d. Total time 76d.
**Phenotype**	Several pairs of ZFN-isogenic hiPSC/hESCs were generated and characterized for neuronal differentiation: hESC – $hESC^{SNCA\ A53T/wt}$, hESC - $hESC^{SNCA\ E46K/wt}$, hiPSC SNCA p.53T – hiPSC corrected.<br>*SNCA* p.A53T was inserted into *SNCA* gene via ZFN without drug selection. Increased efficiency of introducing a second mutation (*SNCA* p.E46K) via single-stranded oligodeoxynucleotides into hESCs. *SNCA* wild-type sequence containing donor vector and ZFNs genetically corrected *SNCA* p.A53T mutation in patient-derived hiPSCs.
**(Ryan et al., 2013)**	
**iPSC Source**	2 isogenic pairs: iPSCs (Soldner et al., 2011) (*SNCA* p.A53T and paired mutation ZFN-corrected clone); hESC (BG01) line and paired ZFN-induced SNCA p.A53T mutation
**iPSC Reprogramming/Number of clones**	Lentivirus KOS, (Dox inducible, Cre-excisable)[8]/6 clones each for p.A53T and isogenic controls
**Gene Editing**	Correction of mutation A53T iPSC; introduction of mutation in hESC by ZFN[8]
**iPSC Maintenance/Passaging**	On HFF in hESC medium +20% KSR, +bFGF[12] / mechanical passaging
**Neural Induction**	iPSC colony dissociation into monolayer, feeder-free culture, enzymatic pass. (Coll, Disp). Induction by KSR media with LDN193189, SB4315423[3], Shh, C25II, Puromorphamine, FGF8 for 5d /2 clones/genotype.
**Neuronal Differentiation/Time**	Shift to N2 medium over 6d. D11 change to Neurobasal/B27, +CHIR. D 13 CHIR replaced by BDNF, GDNF, AA, TGF-β3, cAMP, DAPT for 9d. D20 enzymatic pass. & on PO/L + fibronectin in Neurobasal/B27, +BDNF, GDNF, AA, cAMP, TGF-β3, DAPT. Total time 20d.

**Phenotype**	iPSC-derived dopaminergic neurons from *SNCA* p.A53T carrier show alpha-synuclein aggregation resembling Lewy body-like pathology.<br>*SNCA* p.A53T mutant neurons display variations in mitochondrial machinery and an increase in mitochondrial toxin susceptibility. ROS/RNS abundance leads to changes of MEF2C in *SNCA* p.A53T neurons.
***SNCA* Risk Variants and Gene Regulation**	
**(Soldner et al., 2016)**	
**iPSC Source**	hiPSC line derived from fibroblast AG20446 (male, PD, 57yrs) and 2 hESCs from Whitehead Institute Center for Human Stem Cell Research and NIH (WIBR3, BG01)
**iPSC Reprogramming/Number of clones**	N/A
**Gene Editing**	CRISPR/Cas9 (delete and insert intron enhancer [1834 bp] elements at rs356168 and rs3756054, NACP-Rep 1 [relative sizes: 257 bp, 259 bp, 261 bp, 263 bp] insertion). Lentiviral DOX-induced transgene expression
**iPSC Maintenance/Passaging**	iMEFs in hESC media (DMEM/F12, FBS, KSR, Glu, NEAA, β-ME, FGF2), passaged manually or enzymatically (collagenase type IV)
**Neural Induction**	iPSCs separated from MEFs, cultured in suspension in EB media (DMEM supp. KSR, Glu, NEAA, β-ME, hrNoggin, dorsomorphin) for 8d. EBs plated on poly-L-ornithine/laminin/fibronectin in N2 medium (supp. hrNoggin, dorsomorphin, FGF2). Rosettes microdissected out with trypsin, expanded on poly-L-ornithine/laminin/fibronectin in N2 media with FGF2.
**Neuronal Differentiation/Time**	NPCs passaged 2-4 times, media switched to N2 media supplemented with ascorbic acid, neurons terminally differentiated between day 25 and 31. Total time 25-31d.
**Phenotype**	Generation of CRISPR-modified isogenic hESC allelic panels for *SNCA* gene risk variants rs356168 and NACP-Rep-1. CRISPR insertion of G-allele at rs356168 results in increased expression of *SNCA*. Sequence-specific binding of TFs EMX2 and NKX6-1 represses intron 4 enhancer activity, modulating *SNCA* expression.<br>Allelic series of NACP-Rep1 (genotypes 257/261, 259/261 261/261 263/261) did not show expression differences for alpha-synuclein.
**(Heman-Ackah et al., 2016)**	

**iPSC Source**	Skin fibroblasts: a patient with *SNCA* triplication (ND34391G, iPSCs from NINDS/Coriell Institute), 1 control (NCRM-5, RUDCR Infinite Biologics)
**iPSC Reprogramming/Number of clones**	N/A
**Gene Editing**	CRISPRi-dCas9 targeting SCNA exon 1, 2, and 4.
**iPSC Maintenance/Passaging**	On iMEFs in DMEM/F12 (20% KSR, GlutaMax, MEM NEAA, 0.1 mM β-ME, 10 ng/mL bFGF), passaged manually. For feeder-free, passaged with collagenase IV on Matrigel coated plates in E8 media with ROCK inhibitor.
**Neural Induction**	Control NSCs derived by NIH CRM via PSC Neural Induction Medium and maintained in StemPro NSC SFM (kDMEM, StemPro Neural Supplement, GlutaMax, 20 ng/mL EGF, 20 ng/mL bFGF) SNCA-tri NSCs derived by Applied StemCell NSC Generation Service and maintained in Applied StemCell NSC Expansion Medium (1:1 Neurobasal and DMEM, GlutaMax, N2 supplement, 20 ng/mL bFGF). All NSCs grown on Geltrex.
**Neuronal Differentiation/Time**	Cell suspension seeded on poly-L-ornithine, day 1 media replaced with Neuronal Differentiation Medium (Neurobasal, B27, GlutaMax, BDNF, GDNF), media replaced every other day x7d. Following transfection, cells plated on poly-ornithine/laminin in Neuronal Differentiation Medium (w/ 10 μM ROCK inhibitor and 0.5 Mm dbcAMP). Total time 7d post NSCs.
**Phenotype**	Binding affinity between different sgRNAs and relative position to the TSS are critical for CRISPRi. dCas9 can be used for gene expression manipulations and gene contributions of neurodegenerative disease.<br>CRISPR/dCas9-KRAB and TSS2-1 sgRNA expression reduced endogenous alpha-synuclein mRNA levels in SNCA-tri iPSC-derived neurons by 40%.
**(Tagliafierro et al., 2017)**	
**iPSC Source**	IPSCs from healthy patient (GM23280, Coriell Repository), iPSCs from *SNCA*-tri patient (ND34391, NINDS Repository)
**iPSC Reprogramming/Number of clones**	N/A
**Gene Editing**	N/A
**iPSC Maintenance/Passaging**	On feeder-free Matrigel coated plates in mTeSR media, passaged enzymatically

**Neural Induction**	mDA: EBs formed on AggreWells in NIM with Y27632, plated on Matrigel 5 days later, 1 day later SHH supplemented. Day 12 rosettes selected via Neural Rosette Selection Reagent and put on Matrigel plates with N2B27 media (supp. CHIR99021, SB431542, BSA, bFGF, EGF)<br>BFCNs: EBs formed on AggreWells in NIM. Day 5 EBs replated and media changed daily. Day 8-12 neural rosettes grown in NEM (7 parts KO-DMEM, 3 parts F12, GlutaMax, P/S, B27, FGF, EGF, heparin, SB431542, Y27632). Rosettes selected via NRSR and replated in NEM on Matrigel containing SB431542 and Y27632 for 10d.
**Neuronal Differentiation/Time**	mDA: NPCs passaged onto poly-L-ornithine/laminin in N2B27 media (FGF8, purmorphamine, db-cAMP, L-AA) x14d. Day 14 on, cells fed Maturation Media (GDNF, BDNF, DAPT, db-cAMP, L-AA) and changed every other day.<br>BFCNs: Y27632 withdrawn on day 23, cells plated on poly-L-ornithine/laminin in BrainPhys Neuronal Medium (supp. N2, B27, BDNF, GDNF, L-AA, and db-cAMP), media changed every other day until day 45-50. Total time: mDA:14d, BFCNs: 45-50d.
**Phenotype**	Differentiation into two different neuronal cell types, midbrain dopaminergic and cholinergic neurons, were developed. MiR-7-5p, miR-153-3p, and miR223-3p had higher levels in dopaminergic neurons while miR-140-3p was only slightly increased in cholinergic neurons. SNCA-tri miR-7-5p levels in neurons were 10-fold decreased compared to control neurons, other miRNAs showed similar trends as in control neurons.

References to Supplementary Table 1.

Byers, B., Cord, B., Nguyen, H.N., Schüle, B., Fenno, L., Lee, P.C., et al. (2011). SNCA triplication Parkinson's patient's iPSC-derived DA neurons accumulate alpha-synuclein and are susceptible to oxidative stress. *PLoS One* 6(11)**,** e26159. doi: 10.1371/journal.pone.0026159.

Chung, C.Y., Khurana, V., Auluck, P.K., Tardiff, D.F., Mazzulli, J.R., Soldner, F., et al. (2013). Identification and rescue of alpha-synuclein toxicity in Parkinson patient-derived neurons. *Science* 342(6161)**,** 983-987. doi: 10.1126/science.1245296.

Devine, M.J., Ryten, M., Vodicka, P., Thomson, A.J., Burdon, T., Houlden, H., et al. (2011). Parkinson's disease induced pluripotent stem cells with triplication of the alpha-synuclein locus. *Nat Commun* 2**,** 440. doi: 10.1038/ncomms1453.

Flierl, A., Oliveira, L.M., Falomir-Lockhart, L.J., Mak, S.K., Hesley, J., Soldner, F., et al. (2014). Higher vulnerability and stress sensitivity of neuronal precursor cells carrying an alpha-synuclein gene triplication. *PLoS One* 9(11)**,** e112413. doi: 10.1371/journal.pone.0112413.

Golbe, L.I., Di Iorio, G., Sanges, G., Lazzarini, A.M., La Sala, S., Bonavita, V., et al. (1996). Clinical genetic analysis of Parkinson's disease in the Contursi kindred. *Ann Neurol* 40(5)**,** 767-775. doi: 10.1002/ana.410400513.

Heman-Ackah, S.M., Bassett, A.R., and Wood, M.J. (2016). Precision Modulation of Neurodegenerative Disease-Related Gene Expression in Human iPSC-Derived Neurons. *Sci Rep* 6**,** 28420. doi: 10.1038/srep28420.

Heman-Ackah, S.M., Manzano, R., Hoozemans, J.J.M., Scheper, W., Flynn, R., Haerty, W., et al. (2017). Alpha-synuclein induces the unfolded protein response in Parkinson's disease SNCA triplication iPSC-derived neurons. *Hum Mol Genet* 26(22)**,** 4441-4450. doi: 10.1093/hmg/ddx331.

Mittal, S., Bjornevik, K., Im, D.S., Flierl, A., Dong, X., Locascio, J.J., et al. (2017). beta2-Adrenoreceptor is a regulator of the alpha-synuclein gene driving risk of Parkinson's disease. *Science* 357(6354)**,** 891-898. doi: 10.1126/science.aaf3934.

Oliveira, L.M., Falomir-Lockhart, L.J., Botelho, M.G., Lin, K.H., Wales, P., Koch, J.C., et al. (2015). Elevated alpha-synuclein caused by SNCA gene triplication impairs neuronal differentiation and maturation in Parkinson's patient-derived induced pluripotent stem cells. *Cell Death Dis* 6**,** e1994. doi: 10.1038/cddis.2015.318.

Reyes, J.F., Olsson, T.T., Lamberts, J.T., Devine, M.J., Kunath, T., and Brundin, P. (2015). A cell culture model for monitoring alpha-synuclein cell-to-cell transfer. *Neurobiol Dis* 77**,** 266-275. doi: 10.1016/j.nbd.2014.07.003.

Ryan, S.D., Dolatabadi, N., Chan, S.F., Zhang, X., Akhtar, M.W., Parker, J., et al. (2013). Isogenic human iPSC Parkinson's model shows nitrosative stress-induced dysfunction in MEF2-PGC1alpha transcription. *Cell* 155(6)**,** 1351-1364. doi: 10.1016/j.cell.2013.11.009.

Soldner, F., Laganiere, J., Cheng, A.W., Hockemeyer, D., Gao, Q., Alagappan, R., et al. (2011). Generation of isogenic pluripotent stem cells differing exclusively at two early onset Parkinson point mutations. *Cell* 146(2)**,** 318-331.

Soldner, F., Stelzer, Y., Shivalila, C.S., Abraham, B.J., Latourelle, J.C., Barrasa, M.I., et al. (2016). Parkinson-associated risk variant in distal enhancer of alpha-synuclein modulates target gene expression. *Nature* 533(7601)**,** 95-99. doi: 10.1038/nature17939.

Tagliafierro, L., Glenn, O.C., Zamora, M.E., Beach, T.G., Woltjer, R.L., Lutz, M.W., et al. (2017). Genetic analysis of alpha-synuclein 3' untranslated region and its corresponding microRNAs in relation to Parkinson's disease compared to dementia with Lewy bodies. *Alzheimers Dement* 13(11)**,** 1237-1250. doi: 10.1016/j.jalz.2017.03.001.

Supplemental Table 2: Functional SSVs/SNPs in the *SNCA* gene

SNPs	Location within *SNCA*	Functional /biological effect	System	Cohort, sample size	Reference
Rep1	5' region	Decrease of mRNA expression with short allele	brain	144 controls (SN, temporal and frontal cortex)	Linnertz et al. 2009[1]
			brain	17 PD, 24 controls	Fuchs et al. 2008[2]
rs2736990	Intron	Increase *SNCA* 112/140 ratio (GG highest ratio)	frontal cortex	117 controls (frontal cortex)	McCarthy et al. 2011[3]
rs356168	Intron	No difference for G-allele for *SNCA* expression	temporal cortex	134 controls (frontal, temporal cortex)	Glenn et al. 2017[4]
		Associated with longer 3'UTR	human cortex	17 PD, 17 ctrl, 16 ALS	Rhinn et al. 2012[5]
		G-allele increase of *SNCA* expression, binding to EMX2 and NKX6-1	iPSC neurons	CRISPR-modified stem cell line	Soldner et al. 2016[6]
rs356165	3' region	Increase *SNCA* 112/140 ratio (GG highest ratio)	frontal cortex	117 controls (frontal cortex)	McCarthy et al. 2011[3]
		Lower mRNA levels	temporal cortex, SN	144 controls (SN, temporal frontal cortex)	Linnertz et al. 2009[1]
		No difference in isoform levels	brain	9 PD, 6 ctrl, PMI 5-82hrs	Cardo et al. 2014[7]
rs356219	3' region	Increase *SNCA* 112/140 ratio (GG highest ratio)	frontal cortex	117 controls (frontal cortex)	McCarthy et al. 2011[3]
		CT associated with higher *SNCA* levels	substantia nigra	17 PD, 24 controls	Fuchs et al. 2008[2]
		TT associated with higher *SNCA* levels	cerebellum	17 PD, 24 controls	Fuchs et al. 2008[2]

		No effect	blood	115 PD, 79 controls	Fuchs et al. 2008[2]
		Lower mRNA levels	temporal cortex, SN	144 ctrl (SN, temporal, frontal cortex)	Linnertz et al. 2009[1]
		CC genotype associated with increased a-syn	plasma	86 PD, 78 controls	Mata et al. 2010[8]
rs11931074	3' region	T allele associated with reduced a-syn levels	serum	110 PD, 136 controls	Hu et al. 2012[9]
		No difference in isoform levels	brain	9 PD, 6 ctrl, PMI 5-82hrs	Cardo et al. 2014[7]
rs17016074	3'UTR	Luciferase assay: minor allele expresses more *SNCA*	luciferase assay	SH-SY5Y	Sotiriou et al. 2009

References to Supplementary Table 2:

1 Linnertz, C. *et al.* Genetic regulation of alpha-synuclein mRNA expression in various human brain tissues. *PloS one* **4**, e7480 (2009).

2 Fuchs, J. *et al.* Genetic variability in the *SNCA* gene influences alpha-synuclein levels in the blood and brain. *FASEB journal : official publication of the Federation of American Societies for Experimental Biology* **22**, 1327-1334, doi:10.1096/fj.07-9348com (2008).

3 McCarthy, J. J. *et al.* The effect of *SNCA* 3' region on the levels of *SNCA*-112 splicing variant. *Neurogenetics* **12**, 59-64, doi:10.1007/s10048-010-0263-4 (2011).

4 Glenn, O. C., Tagliafierro, L., Beach, T. G., Woltjer, R. L. & Chiba-Falek, O. Interpreting Gene Expression Effects of Disease-Associated Variants: A Lesson from *SNCA* rs356168. *Frontiers in genetics* **8**, 133, doi:10.3389/fgene.2017.00133 (2017).

5 Rhinn, H. *et al.* Alternative alpha-synuclein transcript usage as a convergent mechanism in Parkinson's disease pathology. *Nature communications* **3**, 1084, doi:10.1038/ncomms2032 (2012).

6 Soldner, F. *et al.* Parkinson-associated risk variant in distal enhancer of alpha-synuclein modulates target gene expression. *Nature* **533**, 95-99, doi:10.1038/nature17939 (2016).

7 Cardo, L. F. *et al.* Alpha-synuclein transcript isoforms in three different brain regions from Parkinson's disease and healthy subjects in relation to the *SNCA* rs356165/rs11931074 polymorphisms. *Neuroscience letters* **562**, 45-49, doi:10.1016/j.neulet.2014.01.009 (2014).

8 Mata, I. F. *et al. SNCA* variant associated with Parkinson disease and plasma alpha-synuclein level. *Archives of neurology* **67**, 1350-1356, doi:10.1001/archneurol.2010.279 (2010).

9 Hu, Y. *et al.* Variant in the 3' region of *SNCA* associated with Parkinson's disease and serum alpha-synuclein levels. *Journal of neurology* **259**, 497-504, doi:10.1007/s00415-011-6209-4 (2012).

Supplemental Table 3: *SNCA* gene methylation in PD and related disorders

Location/genomic regions	Method	% methylation	Cell type/tissue	PD	Reference
**Brain: *SNCA* gene**					
SNCA promoter and intron 1	Bisulfite sequencing of 10 clones, *SNCA* promoter (-2079/-1507) and SNCA intron 1 (-926/-483)	Significantly fewer methylated CpG sites in PD, DNA at specific positions (8, 12, and 17) within intron 1, located within predicted consensus binding sites of TFs	SN, cortex, putamen	PD vs controls (SN and cortex: 6 cases, 6 controls, putamen: 6 cases, 8 controls)	Jowaed et al. 2010[1]
SNCA intron 1 (CpG-2, 13 CpGs)	Bisulfite sequencing of 20 clones	Only demethylation in SN, not in putamen or anterior cingulate	SN, putamen, anterior cingulate	11 PD, 1 DLB, 8 controls	Matsumoto et al. 2010[2]
SNCA intron 1	Methylation-specific PCR	Significant hypomethylation	cortex	PD, DLB, controls (4 samples each)	Desplats et al. 2011[3]
SNCA promoter (17CpGs) and intron 1 (19 CpGs)	454 GS-FLX-based high-resolution bisulphite sequencing	No significant difference between LBD and controls, average methylation of SNCA promoter 0.2-0.8%, SNCA intron 1 0.5-3% methylation	SN, putamen, cingulate gyrus, temporal contrex, cerebellum	LBD vs controls (15 cases, 6 controls), Braak staging	de Boni et al. 2011[4]
SNCA intron 1	Bisulfite sequencing of 10 clones, pyrosequencing	Minor increase in methylation with aging, no gender difference, methylation was higher in neurons compared to non-neuronal cells	whole brain	36 samples (20 male, 16 females, fetus to 90yrs), FACS-sorted neurons and non-neuronal cells from 5 brain samples	de Boni et al. 2015[5]

Location/genomic regions	Method	% methylation	Cell type/tissue	PD	Reference
SNCA intron 1	Bisulfite sequencing of 23 CpGs, 10 clones	No difference, hypomethylated 3.04% PD vs. 3.17% controls, no change in mRNA or protein expression	substantia nigra	PD vs controls (8 cases, 8 controls)	Guhathakurta et al. 2017[6]
**Brain: Global methylation**					
Global methylation	IHC anti 5-mC, ELISA kit	Hypomethylation in PD and DLB (2-fold by IHC, 30% by ELISA)	cortex	PD, DLB, controls (4 samples each)	Desplats et al. 2011[3]
Global methylation	Infinium Human 450K beadchip	0.6% genes differentially methylated, 317 increased, 2591 decreased	frontal cortex	PD vs controls (5 cases, 6 controls)	Masliah et al. 2013[7]
Global methylation	Infinium Human 450K beadchip	0.8% genes differentially methylated, 476 increased, 3421 decreased	leukocytes	PD vs controls (5 cases, 6 controls)	Masliah et al. 2013[7]
Global methylation	ELISA kit	No difference	cerebellum	PD vs controls (36 cases, 27 controls)	Stoger et al. 2017[8]
Global hydroxymethylation	ELISA kit	2-fold difference (0.25% vs. 0.55%, $p<0.001$)	cerebellum	PD vs controls (36 cases, 27 controls)	Stoger et al. 2017[8]
**Peripheral tissues: *SNCA* gene**					
SNCA CpG-1, CpG-2, and *LRRK2* promoter	Bisulfite-specific PCR-based sequencing	Hypomethylation in PD at CpG-2 (5.9% PD vs. 7.69% ctrl), no difference at CpG-1; *SNCA* mRNA increased in PD, hypomethylation even lower in early-onset PD	leukocytes	PD vs controls (100 cases, 100 controls)	Tan et al. 2014[9]
SNCA intron 1 (13 CpGs)	Bisulfite pyrosequencing	No difference, methylation level 9.17% in PD, 9.97% in controls	leukocytes	PD vs controls (50 cases, 50 controls)	Song et al. 2014[10]
SNCA intron 1 (10 CpGs)	Bisulfite pyrosequencing	Overall mean methylation and CpG 4 site methylation lower in DLB, SNCA126 isoform increased in DLB, total SNCA mRNA expression not changed	leukocytes	DLB vs controls (20 cases, 20 controls)	Funahashi et al. 2017[11]

Location/genomic regions	Method	% methylation	Cell type/tissue	PD	Reference
SNCA intron 1, PARK2 promoter	Methylation-specific PCR, ratio of methylated/ unmethylated band intensity by densitometry	Methylation lower in PD for *SNCA* and PARK2 regions	blood	EOPD PD vs controls (91 cases, 52 controls)	Eryilmaz et al. 2017[12]
**SNP associated with *SNCA* methylation**					
Rep-1, *SNCA* intron 1	Bisulfite sequencing of 451bp fragment, 10 clones	Shorter allele associated with higher methylation, mRNA levels not different	PBMC	PD vs controls (100 cases, 95 controls)	Ai et al. 2014[13]
rs3756063, *SNCA* intron 1	Bisulfite sequencing of 451bp fragment, 10 clones	Decreased methylation with risk allele, mRNA levels not different	blood	PD vs controls (91 cases, 92 controls), Chinese Han	Wei et al. 2016[14]
rs3756063 (CpG19), *SNCA* intron 1	Bisulfite treatment with pyrosequencing (14 CpGs)	Hypomethylation in PD, men; decreased methylation with risk allele (G-allele)	blood	PD vs controls (490 cases, 485 controls)	Schmitt et al. 2015[15]
rs3756063, *SNCA* intron 1	Methylation-sensitive RE digest with qPCR	Decrease of methylation with risk allele	blood	PD vs controls (36 cases, 36 controls)	Pihlstrom et al. 2015[16]
rs3756063, *SNCA* intron 1	Methylation-sensitive RE digest with qPCR	Decrease of methylation with risk allele	cortex	PD, controls pooled (12 cases, 12 controls)	Pihlstrom et al. 2015[16]

References to Supplementary Table 3:

1 Jowaed, A., Schmitt, I., Kaut, O. & Wullner, U. Methylation regulates alpha-synuclein expression and is decreased in Parkinson's disease patients' brains. *The Journal of neuroscience : the official journal of the Society for Neuroscience* **30**, 6355-6359, doi:10.1523/JNEUROSCI.6119-09.2010 (2010).

2 Matsumoto, L. *et al.* CpG demethylation enhances alpha-synuclein expression and affects the pathogenesis of Parkinson's disease. *PloS one* **5**, e15522, doi:10.1371/journal.pone.0015522 (2010).

3 Desplats, P. *et al.* Alpha-synuclein sequesters Dnmt1 from the nucleus: a novel mechanism for epigenetic alterations in Lewy body diseases. *The Journal of biological chemistry* **286**, 9031-9037, doi:10.1074/jbc.C110.212589 (2011).

4 de Boni, L. *et al.* Next-generation sequencing reveals regional differences of the alpha-synuclein methylation state independent of Lewy body disease. *Neuromolecular Med* **13**, 310-320, doi:10.1007/s12017-011-8163-9 (2011).

5 de Boni, L. *et al.* DNA methylation levels of alpha-synuclein intron 1 in the aging brain. *Neurobiology of aging* **36**, 3334 e3337-3334 e3311, doi:10.1016/j.neurobiolaging.2015.08.028 (2015).

6 Guhathakurta, S., Evangelista, B. A., Ghosh, S., Basu, S. & Kim, Y. S. Hypomethylation of intron1 of alpha-synuclein gene does not correlate with Parkinson's disease. *Molecular brain* **10**, 6, doi:10.1186/s13041-017-0285-z (2017).

7 Masliah, E., Dumaop, W., Galasko, D. & Desplats, P. Distinctive patterns of DNA methylation associated with Parkinson disease: identification of concordant epigenetic changes in brain and peripheral blood leukocytes. *Epigenetics* **8**, 1030-1038, doi:10.4161/epi.25865 (2013).

8 Stoger, R., Scaife, P. J., Shephard, F. & Chakrabarti, L. Elevated 5hmC levels characterize DNA of the cerebellum in Parkinson's disease. *NPJ Parkinsons Dis* **3**, 6, doi:10.1038/s41531-017-0007-3 (2017).

9 Tan, Y. Y. *et al.* Methylation of alpha-synuclein and leucine-rich repeat kinase 2 in leukocyte DNA of Parkinson's disease patients. *Parkinsonism & related disorders* **20**, 308-313, doi:10.1016/j.parkreldis.2013.12.002 (2014).

10 Song, Y. *et al.* Pyrosequencing analysis of SNCA methylation levels in leukocytes from Parkinson's disease patients. *Neuroscience letters* **569**, 85-88, doi:10.1016/j.neulet.2014.03.076 (2014).

11 Funahashi, Y. *et al.* DNA methylation changes at SNCA intron 1 in patients with dementia with Lewy bodies. *Psychiatry Clin Neurosci* **71**, 28-35, doi:10.1111/pcn.12462 (2017).

12 Eryilmaz, I. E. *et al.* Epigenetic approach to early-onset Parkinson's disease: low methylation status of SNCA and PARK2 promoter regions. *Neurological research* **39**, 965-972, doi:10.1080/01616412.2017.1368141 (2017).

13 Ai, S. X. *et al.* Hypomethylation of SNCA in blood of patients with sporadic Parkinson's disease. *Journal of the neurological sciences* **337**, 123-128, doi:10.1016/j.jns.2013.11.033 (2014).

14 Wei, Y. *et al.* The rs3756063 polymorphism is associated with SNCA methylation in the Chinese Han population. *Journal of the neurological sciences* **367**, 11-14, doi:10.1016/j.jns.2016.05.037 (2016).

15 Schmitt, I. *et al.* L-dopa increases alpha-synuclein DNA methylation in Parkinson's disease patients in vivo and in vitro. *Movement disorders : official journal of the Movement Disorder Society* **30**, 1794-1801, doi:10.1002/mds.26319 (2015).

16 Pihlstrom, L., Berge, V., Rengmark, A. & Toft, M. Parkinson's disease correlates with promoter methylation in the alpha-synuclein gene. *Movement disorders : official journal of the Movement Disorder Society* **30**, 577-580, doi:10.1002/mds.26073 (2015).

Supplementary Table 4: Transcription factor binding in *SNCA* gene

Transcription factor	Binding in *SNCA* genomic region	Mechanism/Effect	Reference
PARP-1	Binding to Rep-1 allele	EMSA, CHIP showed specific binding of PARP-1 to Rep1. Inhibition of PARP-1's catalytic domain increased the endogenous *SNCA* mRNA levels in SH-SY5Y cells, PARP-1 binding to Rep1 reduced transcriptional activity of *SNCA* promoter/enhancer in luciferase assays	Chiba-Falek 2005[1]
C/EBPb	Multiple predicted binding sites	Overexpression of C/EBP beta caused an increase in expression of alpha-synuclein	Gomez-Santos 2005[2], Brenner 2015[3]
GATA-2	GATA-1 occupies a highly restricted region within intron 1 of *SNCA* gene	Knockdown od GATA-2 in SH-SY5Y cells decrease alpha-synuclein expression by about 50%	Scherzer 2008[4], Brenner 2015[3]
ZSCAN21	Binds to intron 1	Transcriptional activator of *SNCA*	Clough 2009[5], 2011[6], Brenner 2015[3]
ZNF219	Binds to 5' region	Complex regulation of *SNCA* gene	Clough 2009[5], 2011[6]
YY1	3' region SNP rs356219-A binds to YY-1	Alpha-synuclein expression unchanged, but antisense non-coding RNA RP11-115D19.1 is stimulated by YY1. Knockdown of RP11-115D19.1 increases alpha-synuclein expression	Mizuta 2013[7]
p53	p53 binding site "CATG" in murine *SNCA* promoter -970 to -967	Feedback loop between alpha-synuclein and p53, depletion of p53 results in down-regulation of alpha-synuclein	Douplan 2016[8]

References to Supplementary Table 4:

1 Chiba-Falek, O., Kowalak, J. A., Smulson, M. E. & Nussbaum, R. L. Regulation of alpha-synuclein expression by poly (ADP ribose) polymerase-1 (PARP-1) binding to the NACP-Rep1 polymorphic site upstream of the *SNCA* gene. *American journal of human genetics* **76**, 478-492 (2005).

2 Gomez-Santos, C. *et al.* Induction of C/EBP beta and GADD153 expression by dopamine in human neuroblastoma cells. Relationship with alpha-synuclein increase and cell damage. *Brain research bulletin* **65**, 87-95, doi:10.1016/j.brainresbull.2004.11.008 (2005).

3 Brenner, S., Wersinger, C. & Gasser, T. Transcriptional regulation of the alpha-synuclein gene in human brain tissue. *Neuroscience letters* **599**, 140-145, doi:10.1016/j.neulet.2015.05.029 (2015).

4 Scherzer, C. R. *et al.* GATA transcription factors directly regulate the Parkinson's disease-linked gene alpha-synuclein. *Proceedings of the National Academy of Sciences of the United States of America* **105**, 10907-10912 (2008).

5 Clough, R. L., Dermentzaki, G. & Stefanis, L. Functional dissection of the alpha-synuclein promoter: transcriptional regulation by ZSCAN21 and ZNF219. *Journal of neurochemistry* **110**, 1479-1490, doi:10.1111/j.1471-4159.2009.06250.x (2009).

6 Clough, R. L., Dermentzaki, G., Haritou, M., Petsakou, A. & Stefanis, L. Regulation of alpha-synuclein expression in cultured cortical neurons. *Journal of neurochemistry* **117**, 275-285, doi:10.1111/j.1471-4159.2011.07199.x (2011).

7 Mizuta, I. *et al.* YY1 binds to alpha-synuclein 3'-flanking region SNP and stimulates antisense noncoding RNA expression. *Journal of human genetics* **58**, 711-719, doi:10.1038/jhg.2013.90 (2013).

8 Duplan, E., Giordano, C., Checler, F. & Alves da Costa, C. Direct alpha-synuclein promoter transactivation by the tumor suppressor p53. *Molecular neurodegeneration* **11**, 13, doi:10.1186/s13024-016-0079-2 (2016).

Supplemental Table 5: Positions/coordinates for UCSC genome browser custom tracks

Figure 1A

**Genome Browser Custom Tracks (GRCh37/hg19)**	**References**
**SNCA Triplications**	
browser position chr4:89584300-90759447	
track name="SNCA Triplications" description="SNCA Triplications" color=255,0,0	
chr4 90033968 92523370 Olgiati	(Ross et al., 2008; Olgiati et al., 2015)
chr4 90165429 92523370 Ibanez	(Ibanez et al., 2004; Ross et al., 2008)
chr4 90645250 92523370 Sekine	(Ross et al., 2008; Sekine et al., 2010)
chr4 90645250 90759447 Keyser	(Ross et al., 2008; Keyser et al., 2010)
chr4 90302002 91143727 Lister	(Fuchs et al., 2007; Ross et al., 2008)
**SNCA Duplications**	
browser position chr4:88343728-94751142	
track name="SNCA Duplications" description="SNCA Duplications" color=0,0,255	
chr4 88343728 94751142 Kara	(Kara et al., 2014)
chr4 88394488 91760188 FPD-321	(Ibanez et al., 2004)
chr4 90645250 92523370 FPD-410	
chr4 90645250 90875780 Jap-A	(Nishioka et al., 2006)
chr4 90645250 90875780 Jap-B	
chr4 90645250 90763144 FPD-437	(Ibanez et al., 2004)
chr4 90645250 92523370 Elia-A	(Elia et al., 2013)
chr4 87870821 91760188 Elia-B	
chr4 86936276 92523370 FPD-131	(Ibanez et al., 2004)
chr4 88529681 94693649 Ikeuchi	(Ikeuchi et al., 2008)
chr4 90645250 90875780 Jap-E	(Nishioka et al., 2006)
chr4 89428083 90875780 Kojovic	(Kojovic et al., 2012)

chr4 88874976 92527977 Sironi	(Sironi et al., 2010)
chr4 90302002 91143727 Lister	(Fuchs et al., 2007; Ross et al., 2008)
**SNCA Trip/Dup**	
browser position chr4:89322975-91310633	
track name="SNCA Dup/Trip" description="SNCA Duplication/Triplication" itemRgb=On	
chr4 90013153 91310633 Italian-Dup 0 . 90013153 91310633 0,0,255	(Ferese et al., 2015)
chr4 90500031 90851296 Italian-Trip 0 . 90500031 90851296 255,0,0	
chr4 89322975 91059278 Iowa-Dup 0 . 89322975 91059278 0,0,255	(Singleton et al., 2003)
chr4 89337388 91047146 Iowa-Trip 0 . 89337388 91047146 255,0,0	
**SNCA Deletions**	
browser position chr4:85839771-98235479	
track name="SNCA Deletions" description="SNCA Deletions" color=0,160,0	
chr4 85839771 93071150 Deletion-2	(ClinVar 2018; Var.ID: 396146)
chr4 89891197 98235479 Deletion-3	(ClinVar 2018; Var.ID: 443904)
chr4 86370518 94894345 Deletion-4	(ClinVar 2018; Var.ID: 152923)
chr4 90167781 91166787 nsv1012406	(Coe et al., 2014)
chr4 90168566 91165346 nsv1323206	(Duyzend et al., 2016)
chr4 90272120 91156917 nsv949454	(Vulto-van Silfhout et al., 2013)
chr4 90458652 91213084 nsv529189	(Miller et al., 2010)

Figure 1B

**Genome Browser Custom Tracks (GRCh37/hg19)**	**References**
**Small SSV and repeats**	
browser position chr4:90742421-90767305	
track name=Rep1-allele description=" " color=163,0,190	
chr4 90767039 90767305 Rep1-allele	(Maraganore et al., 2006)
track name="CT-rich repeat" description=" "	
chr4 90742421 90742492 CT-RichRepeat	(Lutz et al., 2015)
track name="poly T-allele" description=" " color=0,160,0	

chr4 90749444 90749566 PolyT-allele	(Beyer et al., 2007)
**SNPs from GWAS**	
browser position chr4:90759047-90635020	
track name=SNPs description="SNPs" itemRgb=On	
chr4 90758945 90758945 rs2301134 0 . 90758945 90758945 0,0,0	Reviewed in (Campelo and Silva, 2017)
chr4 90759047 90759047 rs2619363 0 . 90759047 90759047 0,0,0	
chr4 90759887 90759887 rs2619364 0 . 90759887 90759887 0,0,0	
chr4 90760828 90760828 rs2583988 0 . 90760828 90760828 0,0,0	
chr4 90678798 90678798 rs2572324 0 . 90678798 90678798 0,0,0	
chr4 90721637 90721637 rs2583959 0 . 90721637 90721637 0,0,0	(Fuchs et al., 2008)
chr4 90678541 90678541 rs2736990 0 . 90678541 90678541 0,160,0	(McCarthy et al., 2011)
chr4 90711770 90711770 rs2737029 0 . 90711770 90711770 0,0,0	Reviewed in (Campelo and Silva, 2017)
chr4 90674431 90674431 rs356168 0 . 90674431 90674431 240,0,0	(Rhinn et al., 2012; Soldner et al., 2016; Glenn et al., 2017)
chr4 90666041 90666041 rs356203 0 . 90666041 90666041 0,0,0	Reviewed in (Campelo and Silva, 2017)
chr4 90675238 90675238 rs3857059 0 . 90675238 90675238 0,0,0	
chr4 90655003 90655003 rs7684318 0 . 90655003 90655003 0,0,0	
chr4 90734535 90734535 rs894278 0 . 90734535 90734535 0,0,0	
chr4 90757505 90757505 rs1372520 0 . 90757505 90757505 0,0,0	
chr4 90657491 90657491 rs3775423 0 . 90657491 90657491 0,0,0	
chr4 90642464 90642464 rs356221 0 . 90642464 90642464 0,0,0	
chr4 90646886 90646886 rs356165 0 . 90646886 90646886 163,0,190	(Linnertz et al., 2009; McCarthy et al., 2011; Cardo et al., 2014)
chr4 90637010 90637010 rs356218 0 . 90637010 90637010 0,0,0	Reviewed in (Campelo and Silva, 2017)
chr4 90637601 90637601 rs356219 0 . 90637601 90637601 163,0,190	(Fuchs et al., 2008; Linnertz et al., 2009; Mata et al., 2010; McCarthy et al., 2011)
chr4 90641340 90641340 rs356220 0 . 90641340 90641340 0,0,0	

chr4 90635020 90635020 rs181489 0 . 90635020 90635020 0,0,0	Reviewed in (Campelo and Silva, 2017)
chr4 90639515 90639515 rs11931074 0 . 90639515 90639515 240,0,0	(Hu et al., 2012; Cardo et al., 2014)
chr4 90757394 90757394 rs3756063 0 . 90757394 90757394 0,0,255	(Pihlstrom et al., 2015; Wei et al., 2016)
chr4 90647278 90647278 rs17016074 0 . 90647278 90647278 240,0,0	(Westerlund et al., 2008; Sotiriou et al., 2009)
chr4 90647640 90647640 rs10024743 0 . 90647640 90647640 0,0,0	Reviewed in (Campelo and Silva, 2017)
chr4 90705364 90705364 rs356186 0 . 90705364 90705364 0,0,0	
**miRNA Binding Sites in *SNCA* 3'UTR**	
browser position chr4:90647024-90647653	
track name="miRNA Binding Sites" description="miRNA Binding Sites"	
chr4 90647314 90647320 miR-153-3p	(Doxakis, 2010)
chr4 90647653 90647659 miR-7-5p	
chr4 90647550 90647556 miR-140-3p	(Tagliafierro et al., 2017)
chr4 90647133 90647140 miR-223-3p	
chr4 90647640 90647646 miR-34b-3p	(Kabaria et al., 2015)
chr4 90647018 90647024 miR-34c-5p	
**Evolutionary conserved regions in *SNCA* gene**	
browser position chr4:90614642-90791735	
track name="ECR Regions" description="ECR Regions"	
chr4 90614642 90614787 D1	(Sterling et al., 2014)
chr4 90614642 90614787 D2	
chr4 90629790 90630480 D3	
chr4 90636848 90637316 D6	
chr4 90659197 90659350 I2	
chr4 90674661 90675121 I5	
chr4 90675762 90675891 I6	
chr4 90682267 90682378 I8	

chr4 90721509 90721763 I12	
chr4 90785647 90785975 U3	
chr4 90789074 90789786 U4-1	
chr4 90791038 90791735 U4-3	
**CpG Islands of *SNCA* gene**	
browser position chr4:90757302-90758870	
track name="CpG Islands" description="CpG Islands"	
chr4 90758009 90758870 Promoter	(Pihlstrom et al., 2015)
chr4 90757302 90757745 Intron-1	(Eryilmaz et al., 2017)
**TF Binding Sites within *SNCA* gene**	
browser position chr4:90749639-90758212	
track name="TF Binding Sites" description="TF Binding Sites"	
chr4 90758190 90758212 ZSCAN21	(Clough et al., 2009; Brenner et al., 2015)
chr4 90749639 90749651 GATA2	(Scherzer et al., 2008; Brenner et al., 2015)
chr4 90674423 90674438 EMX2/NKX6-1	(Soldner et al., 2016)

References to Supplementary Table 5:

Beyer, K., Humbert, J., Ferrer, A., Lao, J.I., Latorre, P., Lopez, D., et al. (2007). A variable poly-T sequence modulates alpha-synuclein isoform expression and is associated with aging. *J Neurosci Res* 85(7)**,** 1538-1546. doi: 10.1002/jnr.21270.

Brenner, S., Wersinger, C., and Gasser, T. (2015). Transcriptional regulation of the alpha-synuclein gene in human brain tissue. *Neurosci Lett* 599**,** 140-145. doi: 10.1016/j.neulet.2015.05.029.

Campelo, C., and Silva, R.H. (2017). Genetic Variants in SNCA and the Risk of Sporadic Parkinson's Disease and Clinical Outcomes: A Review. *Parkinsons Dis* 2017**,** 4318416. doi: 10.1155/2017/4318416.

Cardo, L.F., Coto, E., de Mena, L., Ribacoba, R., Mata, I.F., Menendez, M., et al. (2014). Alpha-synuclein transcript isoforms in three different brain regions from Parkinson's disease and healthy subjects in relation to the SNCA rs356165/rs11931074 polymorphisms. *Neurosci Lett* 562**,** 45-49. doi: 10.1016/j.neulet.2014.01.009.

ClinVar. Bethesda (MD): National Library of Medicine (US), National Center for Biotechnology Information. Variation ID 396146, Allele ID 383039. Retrieved March 07, 2018, from https://www.ncbi.nlm.nih.gov/clinvar/variation/396146/

ClinVar. Bethesda (MD): National Library of Medicine (US), National Center for Biotechnology Information. Variation ID 443904, Allele ID 437566. Retrieved March 07, 2018, from https://www.ncbi.nlm.nih.gov/clinvar/variation/443904/

ClinVar. Bethesda (MD): National Library of Medicine (US), National Center for Biotechnology Information. Variation ID 152923, Allele ID 162674. Retrieved March 07, 2018, from https://www.ncbi.nlm.nih.gov/clinvar/variation/152923/#supporting-observations

Clough, R.L., Dermentzaki, G., and Stefanis, L. (2009). Functional dissection of the alpha-synuclein promoter: transcriptional regulation by ZSCAN21 and ZNF219. *J Neurochem* 110(5)**,** 1479-1490. doi: 10.1111/j.1471-4159.2009.06250.x.

Coe, B.P., Witherspoon, K., Rosenfeld, J.A., van Bon, B.W., Vulto-van Silfhout, A.T., Bosco, P., et al. (2014). Refining analyses of copy number variation identifies specific genes associated with developmental delay. *Nat Genet* 46(10)**,** 1063-1071. doi: 10.1038/ng.3092.

Doxakis, E. (2010). Post-transcriptional regulation of alpha-synuclein expression by mir-7 and mir-153. *J Biol Chem* 285(17)**,** 12726-12734. doi: 10.1074/jbc.M109.086827.

Duyzend, M.H., Nuttle, X., Coe, B.P., Baker, C., Nickerson, D.A., Bernier, R., et al. (2016). Maternal Modifiers and Parent-of-Origin Bias of the Autism-Associated 16p11.2 CNV. *Am J Hum Genet* 98(1)**,** 45-57. doi: 10.1016/j.ajhg.2015.11.017.

Elia, A.E., Petrucci, S., Fasano, A., Guidi, M., Valbonesi, S., Bernardini, L., et al. (2013). Alpha-synuclein gene duplication: marked intrafamilial variability in two novel pedigrees. *Mov Disord* 28(6)**,** 813-817. doi: 10.1002/mds.25518.

Eryilmaz, I.E., Cecener, G., Erer, S., Egeli, U., Tunca, B., Zarifoglu, M., et al. (2017). Epigenetic approach to early-onset Parkinson's disease: low methylation status of SNCA and PARK2 promoter regions. *Neurol Res* 39(11)**,** 965-972. doi: 10.1080/01616412.2017.1368141.

Ferese, R., Modugno, N., Campopiano, R., Santilli, M., Zampatti, S., Giardina, E., et al. (2015). Four Copies of SNCA Responsible for Autosomal Dominant Parkinson's Disease in Two Italian Siblings. *Parkinsons Dis* 2015**,** 546462. doi: 10.1155/2015/546462.

Fuchs, J., Nilsson, C., Kachergus, J., Munz, M., Larsson, E.M., Schüle, B., et al. (2007). Phenotypic variation in a large Swedish pedigree due to SNCA duplication and triplication. *Neurology* 68(12)**,** 916-922.

Fuchs, J., Tichopad, A., Golub, Y., Munz, M., Schweitzer, K.J., Wolf, B., et al. (2008). Genetic variability in the SNCA gene influences alpha-synuclein levels in the blood and brain. *FASEB J* 22(5)**,** 1327-1334. doi: 10.1096/fj.07-9348com.

Glenn, O.C., Tagliafierro, L., Beach, T.G., Woltjer, R.L., and Chiba-Falek, O. (2017). Interpreting Gene Expression Effects of Disease-Associated Variants: A Lesson from SNCA rs356168. *Front Genet* 8, 133. doi: 10.3389/fgene.2017.00133.

Hu, Y., Tang, B., Guo, J., Wu, X., Sun, Q., Shi, C., et al. (2012). Variant in the 3' region of SNCA associated with Parkinson's disease and serum alpha-synuclein levels. *J Neurol* 259(3), 497-504. doi: 10.1007/s00415-011-6209-4.

Ibanez, P., Bonnet, A.M., Debarges, B., Lohmann, E., Tison, F., Pollak, P., et al. (2004). Causal relation between alpha-synuclein gene duplication and familial Parkinson's disease. *Lancet* 364(9440), 1169-1171.

Ikeuchi, T., Kakita, A., Shiga, A., Kasuga, K., Kaneko, H., Tan, C.F., et al. (2008). Patients homozygous and heterozygous for SNCA duplication in a family with parkinsonism and dementia. *Arch Neurol* 65(4), 514-519. doi: 10.1001/archneur.65.4.514.

Kabaria, S., Choi, D.C., Chaudhuri, A.D., Mouradian, M.M., and Junn, E. (2015). Inhibition of miR-34b and miR-34c enhances alpha-synuclein expression in Parkinson's disease. *FEBS Lett* 589(3), 319-325. doi: 10.1016/j.febslet.2014.12.014.

Kara, E., Kiely, A.P., Proukakis, C., Giffin, N., Love, S., Hehir, J., et al. (2014). A 6.4 Mb Duplication of the alpha-Synuclein Locus Causing Frontotemporal Dementia and Parkinsonism: Phenotype-Genotype Correlations. *JAMA Neurol* 71(9), 1162-1171. doi: 10.1001/jamaneurol.2014.994.

Keyser, R.J., Lombard, D., Veikondis, R., Carr, J., and Bardien, S. (2010). Analysis of exon dosage using MLPA in South African Parkinson's disease patients. *Neurogenetics* 11(3), 305-312. doi: 10.1007/s10048-009-0229-6.

Kojovic, M., Sheerin, U.M., Rubio-Agusti, I., Saha, A., Bras, J., Gibbons, V., et al. (2012). Young-onset parkinsonism due to homozygous duplication of alpha-synuclein in a consanguineous family. *Mov Disord* 27(14), 1827-1829. doi: 10.1002/mds.25199.

Linnertz, C., Saucier, L., Ge, D., Cronin, K.D., Burke, J.R., Browndyke, J.N., et al. (2009). Genetic regulation of alpha-synuclein mRNA expression in various human brain tissues. *PLoS One* 4(10), e7480.

Lutz, M.W., Saul, R., Linnertz, C., Glenn, O.C., Roses, A.D., and Chiba-Falek, O. (2015). A cytosine-thymine (CT)-rich haplotype in intron 4 of SNCA confers risk for Lewy body pathology in Alzheimer's disease and affects SNCA expression. *Alzheimers Dement* 11(10), 1133-1143. doi: 10.1016/j.jalz.2015.05.011.

Maraganore, D.M., de Andrade, M., Elbaz, A., Farrer, M.J., Ioannidis, J.P., Kruger, R., et al. (2006). Collaborative analysis of alpha-synuclein gene promoter variability and Parkinson disease. *Jama* 296(6), 661-670.

Mata, I.F., Shi, M., Agarwal, P., Chung, K.A., Edwards, K.L., Factor, S.A., et al. (2010). SNCA variant associated with Parkinson disease and plasma alpha-synuclein level. *Arch Neurol* 67(11), 1350-1356. doi: 10.1001/archneurol.2010.279.

McCarthy, J.J., Linnertz, C., Saucier, L., Burke, J.R., Hulette, C.M., Welsh-Bohmer, K.A., et al. (2011). The effect of SNCA 3' region on the levels of SNCA-112 splicing variant. *Neurogenetics* 12(1), 59-64. doi: 10.1007/s10048-010-0263-4.

Miller, D.T., Adam, M.P., Aradhya, S., Biesecker, L.G., Brothman, A.R., Carter, N.P., et al. (2010). Consensus statement: chromosomal microarray is a first-tier clinical diagnostic test for individuals with developmental disabilities or congenital anomalies. *Am J Hum Genet* 86(5)**,** 749-764. doi: 10.1016/j.ajhg.2010.04.006.

Nishioka, K., Hayashi, S., Farrer, M.J., Singleton, A.B., Yoshino, H., Imai, H., et al. (2006). Clinical heterogeneity of alpha-synuclein gene duplication in Parkinson's disease. *Ann Neurol* 59(2)**,** 298-309.

Olgiati, S., Thomas, A., Quadri, M., Breedveld, G.J., Graafland, J., Eussen, H., et al. (2015). Early-onset parkinsonism caused by alpha-synuclein gene triplication: Clinical and genetic findings in a novel family. *Parkinsonism Relat Disord* 21(8)**,** 981-986. doi: 10.1016/j.parkreldis.2015.06.005.

Pihlstrom, L., Berge, V., Rengmark, A., and Toft, M. (2015). Parkinson's disease correlates with promoter methylation in the alpha-synuclein gene. *Mov Disord* 30(4)**,** 577-580. doi: 10.1002/mds.26073.

Rhinn, H., Qiang, L., Yamashita, T., Rhee, D., Zolin, A., Vanti, W., et al. (2012). Alternative alpha-synuclein transcript usage as a convergent mechanism in Parkinson's disease pathology. *Nat Commun* 3**,** 1084. doi: 10.1038/ncomms2032.

Ross, O.A., Braithwaite, A.T., Skipper, L.M., Kachergus, J., Hulihan, M.M., Middleton, F.A., et al. (2008). Genomic investigation of alpha-synuclein multiplication and parkinsonism. *Ann Neurol* 63(6)**,** 743-750. doi: 10.1002/ana.21380.

Scherzer, C.R., Grass, J.A., Liao, Z., Pepivani, I., Zheng, B., Eklund, A.C., et al. (2008). GATA transcription factors directly regulate the Parkinson's disease-linked gene alpha-synuclein. *Proc Natl Acad Sci U S A* 105(31)**,** 10907-10912.

Sekine, T., Kagaya, H., Funayama, M., Li, Y., Yoshino, H., Tomiyama, H., et al. (2010). Clinical course of the first Asian family with Parkinsonism related to SNCA triplication. *Mov Disord* 25(16)**,** 2871-2875. doi: 10.1002/mds.23313.

Singleton, A.B., Farrer, M., Johnson, J., Singleton, A., Hague, S., Kachergus, J., et al. (2003). alpha-Synuclein locus triplication causes Parkinson's disease. *Science* 302(5646)**,** 841.

Sironi, F., Trotta, L., Antonini, A., Zini, M., Ciccone, R., Della Mina, E., et al. (2010). alpha-Synuclein multiplication analysis in Italian familial Parkinson disease. *Parkinsonism Relat Disord* 16(3)**,** 228-231. doi: 10.1016/j.parkreldis.2009.09.008.

Soldner, F., Stelzer, Y., Shivalila, C.S., Abraham, B.J., Latourelle, J.C., Barrasa, M.I., et al. (2016). Parkinson-associated risk variant in distal enhancer of alpha-synuclein modulates target gene expression. *Nature* 533(7601)**,** 95-99. doi: 10.1038/nature17939.

Sotiriou, S., Gibney, G., Baxevanis, A.D., and Nussbaum, R.L. (2009). A single nucleotide polymorphism in the 3'UTR of the SNCA gene encoding alpha-synuclein is a new potential susceptibility locus for Parkinson disease. *Neurosci Lett* 461(2)**,** 196-201. doi: 10.1016/j.neulet.2009.06.034.

Sterling, L., Walter, M., Ting, D., and Schüle, B. (2014). Discovery of functional non-coding conserved regions in the alpha-synuclein gene locus. *F1000Res* 3(3)**,** 259. doi: 10.12688/f1000research.3281.2.

Tagliafierro, L., Glenn, O.C., Zamora, M.E., Beach, T.G., Woltjer, R.L., Lutz, M.W., et al. (2017). Genetic analysis of alpha-synuclein 3' untranslated region and its corresponding microRNAs in relation to Parkinson's disease compared to dementia with Lewy bodies. *Alzheimers Dement* 13(11)**,** 1237-1250. doi: 10.1016/j.jalz.2017.03.001.

Vulto-van Silfhout, A.T., Hehir-Kwa, J.Y., van Bon, B.W., Schuurs-Hoeijmakers, J.H., Meader, S., Hellebrekers, C.J., et al. (2013). Clinical significance of de novo and inherited copy-number variation. *Hum Mutat* 34(12)**,** 1679-1687. doi: 10.1002/humu.22442.

Wei, Y., Yang, N., Xu, Q., Sun, Q., Guo, J., Li, K., et al. (2016). The rs3756063 polymorphism is associated with SNCA methylation in the Chinese Han population. *J Neurol Sci* 367**,** 11-14. doi: 10.1016/j.jns.2016.05.037.

Westerlund, M., Belin, A.C., Anvret, A., Hakansson, A., Nissbrandt, H., Lind, C., et al. (2008). Cerebellar alpha-synuclein levels are decreased in Parkinson's disease and do not correlate with SNCA polymorphisms associated with disease in a Swedish material. *FASEB J* 22(10)**,** 3509-3514. doi: 10.1096/fj.08-110148.
